# Effect of Alloying and Phase Segregation on the Stability of High-Entropy Alloys: A Case Study on the Dissolution of Os–Ru–Rh–Ir–Pt Nanoparticles

**DOI:** 10.1021/acsaenm.6c00379

**Published:** 2026-06-11

**Authors:** Tatiana Priamushko, Rebecca K. Pittkowski, Maria Minichova, Attila Kormányos, Valentin Briega-Martos, Luis A. Cipriano, Nicolas Schlegel, Rasmus Rohde, Espen D. Bøjesen, Kirsten M. Ø. Jensen, Jan Rossmeisl, Matthias Arenz, Serhiy Cherevko

**Affiliations:** ⊥ Forschungszentrum Jülich GmbH, Helmholtz Institute Erlangen−Nürnberg for Renewable Energy (IET-2), Cauerstr. 1, 91058 Erlangen, Germany; ∥ Center for High Entropy Alloy Catalysis (CHEAC), Department of Chemistry, 4321 University of Copenhagen, 2100 Copenhagen, Denmark; ¶ Institute of Chemical Reaction Engineering, Friedrich-Alexander-Universität Erlangen−Nürnberg, Egerlandstrasse 3, 91058 Erlangen, Germany; ○ Department of Physical Chemistry and Materials Science, University of Szeged, H-6720 Szeged, Hungary; ∇ Department of Chemistry, Biochemistry and Pharmaceutical Sciences, University of Bern, 3012 Bern, Switzerland; # Center for Sustainable Energy Materials (CENSEMAT) and Interdisciplinary Nanoscience Center, 1006 Aarhus University, 8000 Aarhus, Denmark

**Keywords:** high-entropy alloys (HEAs), electrocatalysis, stability, dissolution

## Abstract

High-entropy alloys (HEAs) have emerged as a class of promising electrocatalysts for energy-conversion reactions. In addition to catalytic activity, stability under the reaction conditions is paramount for practical applications. Understanding the dissolution behavior of these multimetallic, complex nanomaterials is therefore essential. Here, we study the dissolution of Os–Ru–Rh–Ir–Pt alloys of different phase compositions to elucidate the influence of elemental mixing on the stability of the materials. The trends in the elemental dissolution are interpreted through theoretical simulations. Combined with local composition analysis from transmission electron microscopy, we identify how local elemental segregation affects the dissolution behavior of precious metal HEAs.

## Graphical abstract

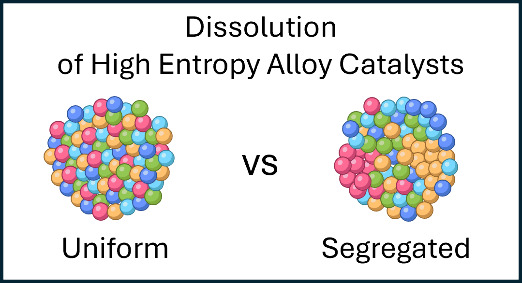

## Introduction

Reducing CO_2_ emissions and transitioning to renewable energy can lessen environmental damage and address the energy crisis. Electrocatalysis is an efficient way to convert and store renewable energy and is at the heart of water and CO_2_ electrolyzers, fuel cells, and metal–air batteries. However, key electrode reactions like oxygen evolution reaction (OER), oxygen reduction reaction (ORR), CO_2_ reduction reaction, and oxidation of alcohols are kinetically slow, requiring high overpotentials and the use of expensive and scarce electrocatalysts. As a result, developing new, active, and affordable catalysts has become a major focus of materials science and electrochemistry. Research has shown that catalytic activity correlates with the binding energies of reaction intermediates,^1–3^ and tuning these energies by incorporating additional elements has enabled the design of diverse multimetallic and hybrid materials.^4–6^

One of the most complex types of recently developed materials used as electrocatalysts are high-entropy alloys (HEAs). HEAs can be described as alloys composed of (1) at least five elements with molar fractions between 5 and 35 atom % or (2) alloys with configurational entropy higher than 1.5*R* (*R* = gas constant).^7^ After their discovery in 2004 and the discovery of their excellent mechanical properties,^8,9^ these materials received significant interest as catalysts and electrocatalysts due to their unique features, such as multifunctionality, elemental composition flexibility, and thermodynamic stability.^10–12^ Thanks to the high-entropy effect (a higher mixing entropy lowers the free energy of the solution and facilitates its formation), combining many elements should create a single-phase solid solution that provides unique active sites, which makes HEAs an attractive platform for materials discovery of promising, highly active electrocatalysts.^5,7,13^ However, the formation of a solid single-phase solution is not always straightforward, and many HEAs investigated in the early years were multiphase alloys rather than solid single-phase solutions.^10,12,14,15^

There are many obstacles in synthesizing and applying HEAs for electrochemical energy conversion. Rationally designing high-entropy materials is very challenging because this research field is still at an early stage.^16–18^ Although HEAs’ functionality can be tuned by altering their composition, the main challenge lies in identifying the influence of individual elements on their properties.^5,7^ Moreover, many studies focused mostly on the strategies to improve only one of the important catalytic properties—activity. To date, one can find literature on HEAs with enhanced activity for both OER and ORR, still, without reported data on their stability, which is a critical factor in determining the applications of materials in many industrial fields.^6,19,20^ Additionally, it is unclear how phase segregation in such complex systems affects the materials’ stability and whether aiming for a solid single-phase solution is required. The stabilizing effect of high entropy in solid single-phase solutions of five or more elements is often taken for granted, in terms of not only the structural stability but also the electrochemical stability. However, this assumption overlooks the fact that the entropy contribution is temperature-dependent, and at room temperature, these materials are, in fact, metastable.^21^ Furthermore, there is a lack of ex situ and especially in situ experimental results backing this assumption.^22,23^

In our previous work, we showed that the high-entropy effect does not always play a role in the electrochemical stability.^24^ This work aims to provide insights into the electrochemical stability of HEAs with different degrees of elemental mixing. We examine the electrochemical stability of various Os–Ru–Rh–Ir–Pt HEAs with different crystallographic phase compositions to uncover the impacts of the phase composition on the alloys’ stability in a wide potential range. We selected alloys based on platinum group metals because the elements are known to be active for various electrocatalytic reactions, as well as reasonably stable in acidic electrolytes.^25–29^ A single-source precursor synthesis was used to vary the crystalline phase formation of the HEA nanoparticles.^15,30^ The Os–Ru–Rh–Ir–Pt element system was chosen based on these prior reports demonstrating that this synthesis enables the formation of single-phase HEA nanoparticles with tunable composition and mixing within this specific set of platinum group elements. The selected element composition is of interest, e.g., for ORR electrocatalysis, motivating its selection alongside its suitability for forming single-phase multicomponent alloys.^30^ The phase-pure and phase-segregated alloys were prepared by changing synthesis conditions and elemental ratios in the precursors. The stability of the materials in an acidic electrochemical environment was assessed by the scanning flow cell combined with inductively coupled plasma mass spectrometry.^31^ This unique in-line technique allows the simultaneous dissolution tracking of multiple elements under various electrochemical conditions.^32^

## Experimental Part

### Synthesis

The HEA nanoparticle samples were synthesized as detailed in a previous study.^15,30^ For the synthesis, commercial precious metal precursors were used as received from the supplier (Sigma-Aldrich): [Ir­(NH_3_)_5_Cl]­Cl_2_, [Rh­(NH_3_)_5_Cl]­Cl_2_, [Ru­(NH_3_)_5_Cl]­Cl_2_·(NH_4_)_2_[IrCl_6_], (NH_4_)_2_[PtCl_6_], and (NH_4_)_2_[OsCl_6_]. In short, separate aqueous solutions of metal hexachlorate precursors and metal chloropentaamines were prepared by dissolving the respective precursor salts in ultrapure water (Milli-Q IQ 7000, 18.2 MΩ·cm, 2.7 ppb TOC) (90 °C). For all syntheses, a total metal concentration of 0.005 mol L^–1^ was used, i.e., concentrations of 2.5 mM hexachlorates and 2.5 mM chloropentaamines. The solutions were stirred with heating to ensure mixing of the metal precursors in the solution. The hot solutions were blended, and a single-source precursor was obtained as the precipitate, which was washed with Milli-Q water and dried before the calcination step. The single-source precursor was reduced in a quartz-tube furnace (Gero SR-A 40-250/11) in 5% H_2_ in Ar (Carbagas) to obtain the final HEA product. The samples were calcined for 60 min with a heating ramp of 50 K min^–1^ at 500 or 700 °C. To obtain the different samples, the ratio of metal salts used, as well as the calcination procedure, was varied. The nominal composition of the precursor solution was Os_0.10_Ru_0.10_Rh_0.27_Ir_0.27_Pt_0.27_ for the face-centered-cubic (fcc)-dominated samples fcc-HEA (calcination at 700 °C) and 2fcc-HEA (calcination at 500 °C). The sample hexagonal-close-packed hcp-2fcc-HEA was prepared with a nominal composition of Os_0.10_Ru_0.10_Rh_0.25_Ir_0.3_Pt_0.25_ and calcined at 700 °C.

### Characterization

#### Nanomaterial Structure

The crystalline powders obtained from the calcination procedures were characterized by powder X-ray diffraction (XRD) data using a STOE StadiP with a Cu Kα X-ray source in transmission geometry. To determine the phase fraction of the hcp and fcc phases present, Rietveld refinements of the XRD patterns of the powder samples were performed with the FullProf suite program package.^33^ Rietveld refinements were performed with the scale factor, lattice parameters, *B*_iso_ values, and peak-shape parameters refined, while site occupancies were fixed. Os was used to represent the atomic position in all refinements because its atomic number is closest to the average *Z* of the five constituent elements. Models of the metallic close-packed lattices were fit to the data: fcc (*Fm*3̅*m*, No. 225) or hcp (*P*63/*mmc*, No. 194). The overall elemental composition was estimated by energy-dispersive X-ray spectroscopy (EDX) using a Zeiss Gemini 450 scanning electron microscope equipped with an Oxford Instruments UltimMax 65 EDX detector.

#### High-Resolution Transmission Electron Microscopy (TEM)

Bright-field TEM images were collected using a Talos FX-200 equipped with a Ceta 16 M camera with samples mounted in a single tilt holder. STEM images were collected with the same microscope. A 10.5-mrad-converged electron probe was used. The collection angle of the high-angle annular dark-field STEM images was 60–200 mrad. Scanning transmission electron microscopy combined with energy-dispersive X-ray spectroscopy (STEM–EDX) data sets were collected using the ChemiSTEM system installed on the microscope. The STEM–EDX data were binned (either 4 × 4 or 6 × 6), and background subtraction via linear interpolation around the relevant elemental peaks was performed before plotting the maps reflecting the spatial distribution of the different elements using the HyperSpy^2^ python library.

#### Electrochemical Characterization

In the preparation for cyclic voltammetry (CV) measurements, PTFE-embedded glassy carbon (GC) disks with a diameter of 5 mm were mechanically polished on a polishing cloth (MicroCloth, Buehler) for 2 min using an alumina slurry (0.3 μm MicroPolish, Buehler). Then, the tips were rinsed in ultrapure water, placed in a mixture of 2-propanol (VWR, 99.8%) and ultrapure water, sonicated for 15 min in a sonication bath, and subsequently rinsed with ultrapure water again. The as-prepared samples were dispersed in a 3:1 mixture of ultrapure water and 2-propanol to yield a final concentration of 0.5 mg_sample_ mL^–1^. A horn sonicator (Qsonica sonicator, Q500) was used to obtain a homogeneous dispersion, during which the sample was cooled in an ice bath to prevent solvent evaporation. A total of 7.84 μL of the dispersion was then pipetted onto the polished GC disks, which were subsequently dried under a humidified N_2_ stream. This resulted in a catalyst loading of 20 μg cm^–2^. An Au coil served as the counter electrode. Applying a current of −20 mA to a Pt wire situated in a glass tube produced the trapped hydrogen electrode (RHE), which served as the reference electrode. A custom-built one-compartment glass cell was used to conduct all experiments. Before the experiments, it was cleaned by boiling it in 25% HNO_3_, followed by thorough rinsing with ultrapure water, and finally boiling it therein as well. A total of 100 mL of Ar-purged 0.1 M HClO_4_ (70% Suprapur, Merck) served as the electrolyte solution. A prepared sample tip, serving as the working electrode, was mounted onto a EDI101 rotating disk electrode assembly (Hach-Lange). An ECi-210 potentiostat controlled by the EC4 DAQ 4.2 software (both Nordic Electrochemistry ApS) was used to control the electrical current and potential. All CV curves were recorded at a scan rate of 25 mV s^–1^. The lower vertex was fixed at 0.05 V_RHE_, whereas the upper vertex was varied from 0.8 to 1.5 V_RHE_ in 0.1 V steps. At each distinct upper vertex potential, five CV curves were recorded. An alternating-current perturbation with a frequency of 5000 Hz and an amplitude of 5 mV was superimposed during the measurements to determine the effective solution resistance. The measured resistance values were used to correct the *iR* drop.

#### Online Inductively Coupled Plasma Mass Spectrometry (ICP-MS) Measurements

Suspensions of HEAs were prepared with ultrapure water and 2-propanol in a ratio of 7:1. Nafion (Sigma-Aldrich, 5 wt %) was added to the suspension as a binder. The suspension was sonicated with a sonication horn (Branson SFX 150) for around 30 min with intervals (4 s pulse and 2 s pause) and 40% intensity until the ink was homogeneous. To prevent heating of the ink mixture, the vial was kept on ice during sonication. After that, 0.25 μL of the suspension was drop-casted onto a GC plate, serving as a working electrode. The loading of the catalysts was aimed to be 20–25 μg cm^–2^. The quality and area of the drop-casted spots were examined using an optical microscope (Keyence VK-X250). The electrochemical stability of the drop-casted samples was examined with a scanning flow cell combined with ICP-MS.^31^ A GC rod and a Ag/AgCl electrode (Metrohm) were used as counter and reference electrodes, respectively. Freshly prepared 0.1 M HClO_4_ (70% Suprapur HClO_4_, Merck), saturated with Ar, was used as the electrolyte and purged through the setup with a flow rate of 3.50 ± 0.05 μL s^–1^. The electrolyte flow rate was controlled by the peristaltic pump of the ICP-MS instrument (Elemental Scientific M2) and calibrated daily. The working electrode was placed on a translational stage (Physik Instrumente M-403) that allows it to quickly move along the electrode and rapidly screen multiple samples. All electrochemical measurements were performed using a Gamry Reference 600 potentiostat. All instruments (gas control box, mass flow controllers, peristaltic pump, and translational stage) were controlled by homemade LabView software. The electrocatalytic stability of the materials was investigated by applying three electrochemical protocols. Protocol A included a 5 min potentiostatic hold at 0.05 V_RHE_, which was followed by three CV cycles with different upper potential limits (UPL = 0.9, 1.2, and 1.5 V_RHE_) and a scanning rate of 5 mV s^–1^. Protocols B and C were based on the potentiostatic holds at varied potentials: 5 min of hold at 0.05 V_RHE_, 10 min of hold at 1.2 or 1.5 V_RHE_, Protocol B and C, respectively) and 5 min of hold at 0.05 V_RHE_. The ICP-MS instrument (PerkinElmer NexION 350X) was calibrated daily with known amounts of analyzed metals (Os, Ir, Ru, Pt, and Rh) and internal standards (Re^187^, Mo^98^, and In^115^). Both electrochemical and ICP-MS results were normalized by the geometric surface area of the drop-casted catalyst spots measured individually by a laser scanning microscope.

#### Density Functional Theory (DFT) Simulations

To simulate the dissolution of single-phase HEA nanoparticles, we study Os–Ru–Rh–Ir–Pt HEA surfaces in the fcc crystal structure. A stable fcc phase forms when a large fraction of fcc-forming metals (here: Rh, Ir, and Pt) is included, which stabilizes the fcc crystal structure.^15^ The surface composition was chosen to resemble the elemental composition of the single-phase fcc HEA nanoparticles formed experimentally. In the model, we assumed that nanoparticles have different exposed surfaces and each surface has different types of atoms with varying coordination numbers (cn). For instance, we have considered two surfaces with low coordination atom environments because, in previous studies, we have seen that such atoms are more prone to dissolution and often dominate the early stage of this process:^34,35^ kink atoms with cn = 6 by using a kink surface generated from the step-edge (211) surface (Figure S2) and edge atoms with cn = 7 by using the step-edge (211) surface (Figure S2). First, we have optimized the bulk structure of each metal: Os, Ru, Rh, Ir, and Pt. Subsequently, hundreds of random Os–Ru–Rh–Ir–Pt HEA surfaces with almost the same experimental composition of the fcc-HEA were generated (kink, Os_0.08_Ru_0.10_Rh_0.26_Ir_0.20_Pt_0.36_, edge, Os_0.06_Ru_0.10_Rh_0.26_Ir_0.23_Pt_0.36_). To generate each Os–Ru–Rh–Ir–Pt HEA surface, we used the average lattice parameter derived from the DFT-optimized bulk structures of each constituent metal. The procedure to calculate the theoretical onset potential is reported in our previous study,^34^ and the standard electrode potentials used for each element were taken from the Pourbaix diagrams (Table S1).^36^ The DFT calculations were performed with the GPAW code,^37^ and the atomic structures were manipulated with ASE.^38^ For all calculations, the revised Perdew–Burke–Ernzerhof exchange-correlation functional was implemented.^39^ The electron wave functions were treated with periodic plane-wave functions with an energy cutoff of 400 eV. The constructed structures correspond to 3 × 3 supercells with four atomic layers with periodic boundary conditions in the lateral directions and a vacuum layer of 10 Å along the *z* direction. The forces on the atoms were relaxed until the maximum force on any atom was below 0.05 eV Å^–1^.

## Results and Discussion

### Phase Segregation and Elemental Mixing in the HEAs

Multimetallic nanoparticles of five precious metals (Os, Ru, Rh, Ir, and Pt) were prepared following a literature procedure of calcining single-source precursors in reductive gas atmospheres.^15^ By changing the synthesis parameters, namely, the precursor ratios and reduction temperature, a set of samples with different elemental and phase compositions was obtained. This allows us to investigate the effect of phase segregation on the stability of HEAs while conducting electrochemical protocols. Synthesis details are given in the Experimental Part.

Three samples with similar elemental compositions but containing different crystallographic phases were prepared. The XRD patterns of the HEAs are shown in Figure 1a,b, along with the theoretical diffraction pattern of a Pt reference. To analyze the atomic distribution of all elements in the samples, STEM–EDX analysis was used. The elemental distribution maps created from STEM–EDX measurements are shown in Figure 1c–e. To highlight elemental segregation, additional Ir and Pt composite maps are included. Table 1 shows the overall nominal elemental composition of the prepared samples, the crystallographic phases identified, and lattice parameters determined by the Rietveld refinement of the XRD patterns, and summarizes the nanoscale compositional heterogeneity of the samples based on the STEM–EDX data analysis. The phase composition as well as local elemental segregation and intermixing are discussed in detail below for each sample.

**Figure 1:**
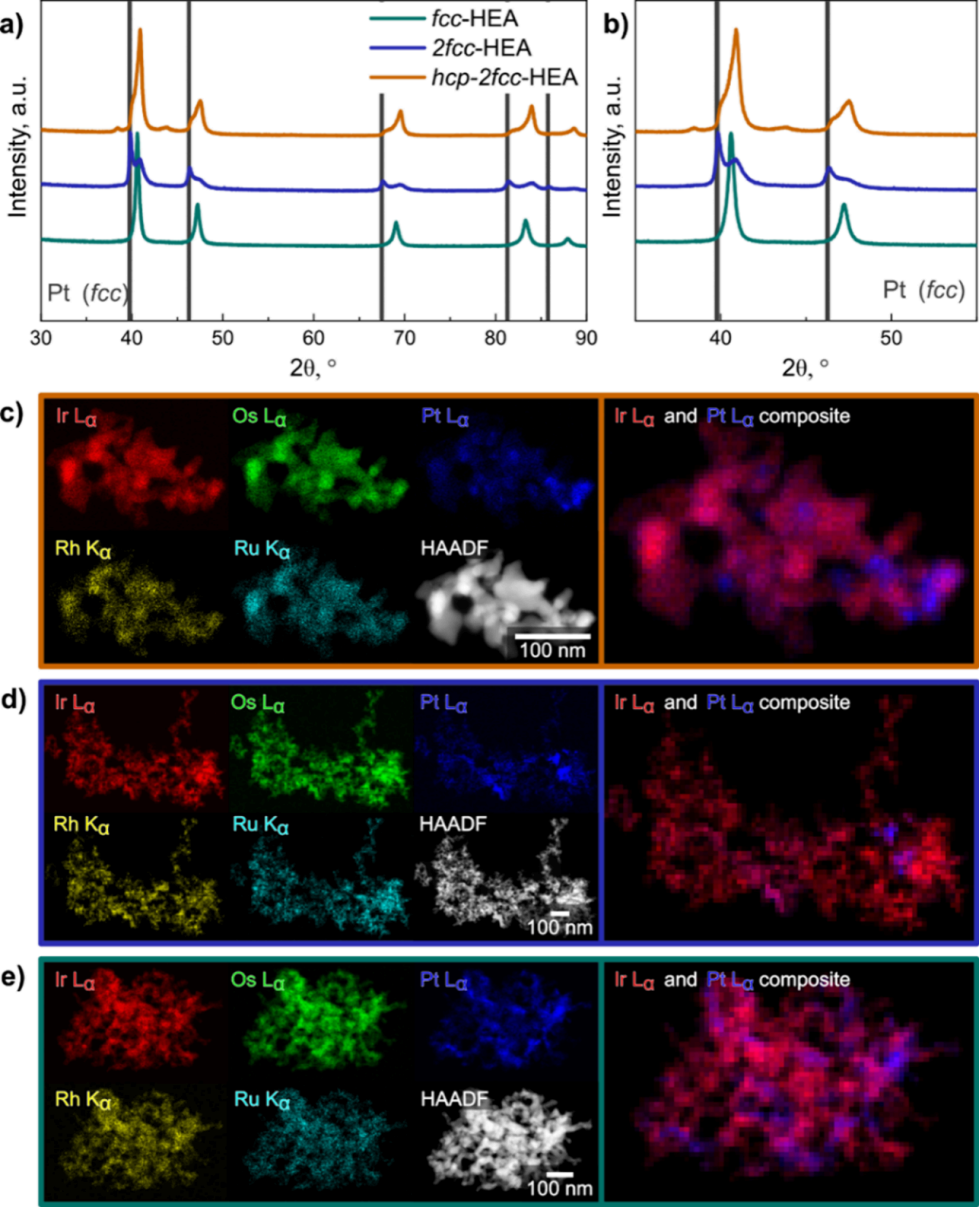
(a) XRD patterns of HEAs with (b) zoom-in on the low 2θ angles where the phase separation is most pronounced. High-resolution STEM–EDX mapping of the three samples (c) fcc-HEA, (d) 2fcc-HEA, and (e) hcp-2fcc-HEA with additional Ir and Pt composited maps, highlighting areas of high Ir and Pt content.

**Table 1:** Average Elemental Composition Determined by STEM–EDX Analysis, Crystalline Phase Composition Determined from the Rietveld Refinement of XRD Patterns, and Summary of the Nanoscale Compositional Heterogeneity as Indicated by NMF Analysis of the STEM–EDX Data

sample	Os, atom %	Ru, atom %	Rh, atom %	Ir, atom %	Pt, atom %	phases	lattice parameter, Å	nanoscale compositional heterogeneity
fcc-HEA	6	12	29	20	32	fcc	3.8458(1)	all elements well-distributed, minor segregation of Ir and Pt
2fcc-HEA	7	11	28	22	30	fcc	3.8332(3)	local segregation of Os and Ru
						fcc	3.9202(2)	separation of Pt
hcp-2fcc-HEA	3	18	23	37	18	fcc	3.8294(2)	Ru- and Os-rich areas
						fcc	3.8792(4)	Pt and Ir minor segregated
						hcp	*a* = 2.7059(1), *c* = 4.3770(5)	

One sample presents a single crystallographic phase (green diffraction pattern in Figure 1a,b). The structure of this single-phase alloy can be described as face-centered-cubic (fcc). We denote this sample, therefore, as fcc-HEA in the following. Table S2 and Figure S3 contain the results of the Rietveld refinement, which can be well-described by the presence of only one phase in this sample. The refined lattice parameter *a* of 3.8458(1) Å agrees with the lattice constant expected for a single-phase fcc alloy of the composition determined by EDX analysis.^30^ Moreover, the elemental mapping (Figures 1e and S4) does not depict any major inhomogeneities in the distribution of elements throughout the sample.^30^ While all elements seem to be well distributed throughout the particles, Ir and Pt tend to exhibit local segregation. Nonnegative matrix factorization (NMF) analysis was used to identify elemental clustering at the nanometer scale, revealing patterns not directly discernible to the human eye.^30^ The results are shown in Figure S5. The NMF algorithm identifies three components. This includes two factors that are well-mixed with each other: one containing all elements but rich in Pt and one Ir-rich and Pt-deficient component. NMF analysis also identifies a third Os- and Rh-rich component without Pt and Ir, which appears to be less mixed with the other two factors. Estimations of the atomic percentages of the NMF components were found by Cliff–Lorimer quantification but exhibit high uncertainty and are therefore only shown in the Supporting Information (SI). NMF analysis thus confirms that, while all elements are well-distributed, Ir and especially Pt exhibit reduced mixing at the local level.

We further prepared two samples of comparable elemental composition that contain multiple crystallographic phases. One sample consists of two fcc phases of different lattice constants (blue diffraction pattern in Figure 1a,b). We call this sample 2fcc-HEA. The Bragg peaks of the more crystalline phase agree well with those of a Pt reference, as shown in black in Figure 1a,b, indicating the presence of pure Pt metal. On the basis of the Rietveld refinement, we find that sample 2fcc-HEA contains around 40 wt % of an fcc phase with a lattice parameter of *a* = 3.9202(2) Å, agreeing with Pt (Table S2). For the second fcc phase, the lattice parameter was estimated as *a* = 3.8332(3) Å, which we attribute to an HEA phase of different elemental composition. The slightly smaller lattice parameter of 3.8332 Å compared to that of fcc-HEA (3.8458 Å) agrees with an fcc phase containing less Pt. The elemental mapping (Figure 1d) shows that Pt is only present in separate, localized Pt-rich areas, which corroborates that the second fcc phase of 2fcc-HEA is a platinum side phase. Aside from Pt-enriched parts, the other areas appear to contain all elements in a well-mixed fashion. These areas can be attributed to the HEA fcc phase, which appears to be a solid solution. This HEA phase is thus Pt-deficient compared to the fcc-HEA sample, as is evident from the elemental maps in Figures 1d and S6, which is also reflected in its smaller lattice parameter (*a* = 3.8332 Å). For the selected area of the 2fcc-HEA sample analyzed with STEM–EDX, NMF analysis picks up three components: one without Ru, one without Pt, and one containing only Os and Ru (Figure S7). However, the two essentially Pt-free NMF components are very well blended throughout individual particles, and the diffraction does not indicate a separate hcp phase. We therefore conclude that the segregation of the hcp metals Os and Ru is only very minor and on a local level, while Pt appears to be crystallographically separated and mostly absent from the HEA phase. The formation of two phases, one consisting predominantly of Pt and one a well-mixed OsRuRhIr phase, can be explained by the formation mechanisms of these multielement materials. It has been shown that the individual metal precursors reduce stepwise during the material synthesis, with Pt exhibiting the lowest reduction temperature.^30^ Thus, the separate platinum-based phase can be attributed to the lower calcination temperature of 2fcc-HEA compared to fcc-HEA.

The third HEA sample is characterized by the presence of two fcc phases as well as an additional hcp phase. In this hcp-2fcc-HEA sample (orange diffraction pattern in Figure 1a,b), the two fcc phases are of different lattice parameters [*a*_1_ = 3.8294(2) Å, 61 wt %, and *a*_2_ = 3.8792 Å, 25 wt %] and do not indicate clear Pt separation. The additional hcp phase [14 wt %, *a* = 2.7059(1) Å, and *c* = 4.3770(5) Å] points toward a tendency of separation of ruthenium and/or osmium. Here, it is less straightforward to infer the composition of the segregated phases from the elemental mapping (see Figures S8 and S9 and the discussion in the SI) because the elements appear quite well distributed throughout the particles. NMF analysis identifies three components: one rich in Os, Ru, and Rh, while the last two are mainly composed of Pt and Ir, respectively. The first component is most likely the hcp phase because it contains the most Os and Ru. In the Ir/Pt composite maps in Figure 1c, the purple color also indicates that Pt and Ir are less segregated in this sample compared to the other two.

From the diffraction patterns presented in Figure 1a,b, as well as the TEM images (Figure S10), one can see that the crystallite and particle sizes of the samples are similar. The samples’ morphology can be described as randomly shaped, prolonged particles that are interconnected with each other. The size of such interconnected polycrystalline particles is comparable for all samples.

Before studying the dissolution of HEAs under electrochemical conditions, we performed an initial CV test to inspect the general electrochemical behavior of the materials. The recorded CV curves are depicted in the SI, and their general features are discussed (Figure S11). The CV curves have broad features, as expected for compositionally complex multielement samples.

### Dissolution of a Single-Phase fcc-HEA

We first discuss the stability of the single-phase fcc-HEA in an acidic environment under various electrochemical conditions. To investigate the dissolution behavior in a wide potential range, we performed potential cycling with an increasing UPL (Protocol A). The initial potential scan varied from 0.05 to 0.9 V_RHE_ with a scan rate of 5 mV s^–1^, while for the second and third cycles, the potential was increased to 1.2 and 1.5 V_RHE_, respectively. To study anodic (during metal oxidation) and cathodic (during metal oxide reduction) dissolution trends separately, we performed two protocols with potentiostatic holds at different potentials: the initial and final potentials were kept the same for both protocols (0.05 V_RHE_), while the upper potential was changed from 1.2 V_RHE_ (Protocol B) to 1.5 V_RHE_ (Protocol C).

The dissolution profiles for Protocols A and B of the fcc-HEA are depicted in Figure 2a,b, while the dissolution profile of Protocol C is shown in Figure S12a. Osmium is the least stable element, exhibiting dissolution exclusively during the anodic sweep.^40^ Osmium dissolution starts at significantly lower potentials than those of the other noble metals in the alloy (Figures 2a and 3a) and increases rapidly with rising potential. This dissolution process can be attributed to the formation of osmium oxide intermediates (mostly OsO_2_ and OsO_4_), which are soluble in acidic media.^40^ As seen in Protocols B and C, a fast ramp to more positive potentials causes prompt anodic dissolution of Os, which subsequently decreases over time during the potentiostatic hold at 1.2 or 1.5 V_RHE_. The following switch of the potential in the cathodic direction does not result in Os dissolution, which may be due to factors such as depletion of the catalyst surface of osmium and high irreversibility of osmium oxide reduction.

**Figure 2:**
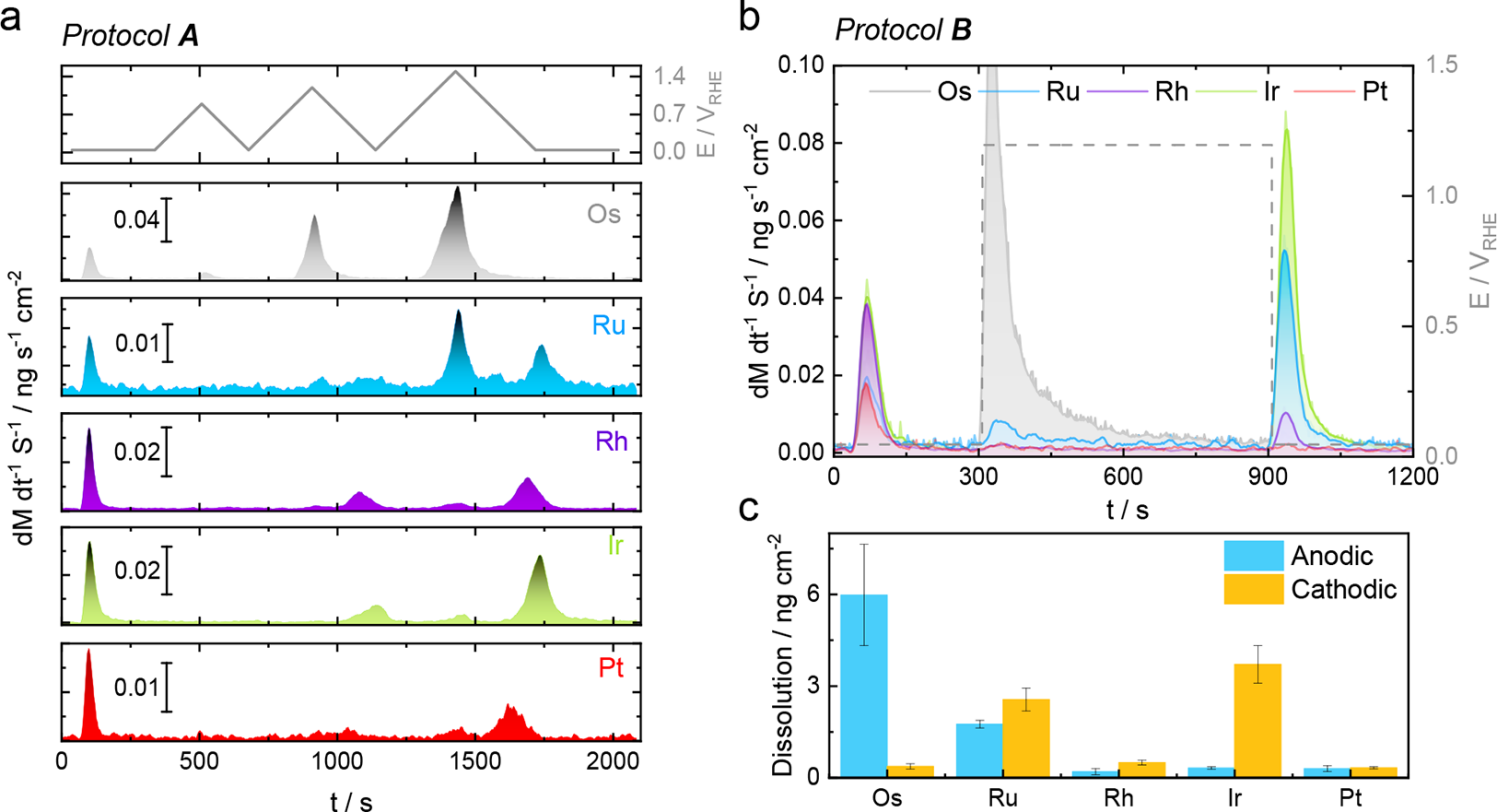
Element-specific dissolution profiles of the fcc-HEA sample as observed using (a) Protocol A and (b) Protocol B. (c) Quantified metal dissolution during Protocol B extracted from the integrated anodic and cathodic dissolution peaks in panel b.

**Figure 3:**
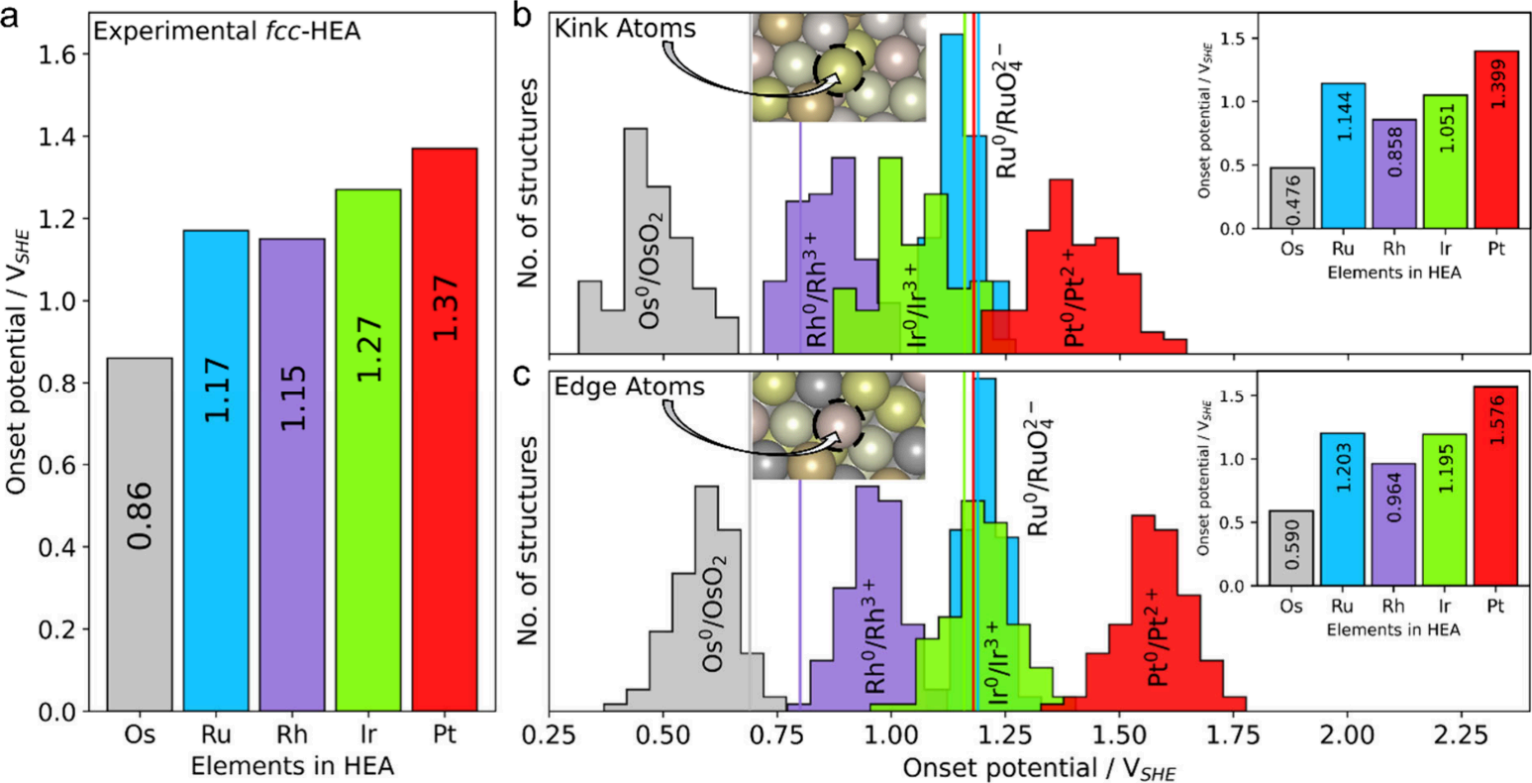
Comparison between the (a) experimental onset potential of the fcc-HEA (Os_0.06_Ru_0.12_Rh_0.29_Ir_0.20_Pt_0.32_) and the potential distribution of dissolving (b) kink and (c) edge atoms from the modeled HEA (kink, Os_0.08_Ru_0.10_Rh_0.26_Ir_0.20_Pt_0.36_; edge, Os_0.06_Ru_0.10_Rh_0.26_Ir_0.23_Pt_0.36_). The vertical lines indicate the dissolution potentials of the pure elements.

Being the state-of-the-art electrocatalyst for the OER, iridium (oxide) has been intensively studied over the last few decades. We have published detailed works on the stability of Ir in an acidic environment under applied potentials.^41–45^ As opposed to osmium, iridium dissolves mostly cathodically, as can be seen in Figures 2 and S12b. According to the mentioned works, the cathodic dissolution of Ir can be explained as the dissolution of the anodically formed oxides during their reduction to metal. Cycling the potential up to different UPLs in Protocol A reveals that the anodic dissolution of Ir in the studied fcc-HEA starts at higher potentials than it commonly starts for pure Ir metal (Figure S13). This may be explained by the lower accessibility of the Ir sites on the catalyst’s surface, leading to a possible shift of redox processes (Ir^3+^/Ir^0^) to higher potentials or the overall lower amount of Ir in the catalyst, resulting in longer times required to reach a concentration above the detection limit of ICP-MS.

In contrast to Ir, a higher anodic dissolution is observed for Ru. Moreover, its onset potential of dissolution is slightly lower compared to Ir, although it is much higher than that of the pure Ru metal (Figure S13).^24,46,47^ A small anodic dissolution peak is observed already during cycling up to 1.2 V_RHE_, corresponding to the formation of various Ru oxides (RuO_*x*_). This is followed by cathodic dissolution, which can be assigned as the reduction of previously formed oxide species. When the potential increases up to 1.5 V_RHE_, the anodic dissolution peak, as well as the following cathodic peak, is clearly defined. Remarkably, in this case, cathodic dissolution is distinct from the behavior observed previously for Ru thin films.^24^ In the fcc-HEA, two cathodic peaks can be observed when the potential is changing negatively: the first low-intensity peak is observed at around 0.75 V_RHE_, and the second, more pronounced peak is observed starting at 0.2–0.1 V_RHE_. In the previously published works on Ru thin films, when cycling to UPL higher than 1.2 V_RHE_, only one anodic dissolution peak of Ru was observed. This was assigned to the constant dissolution of Ru caused by the thermodynamically unstable RuO_4_^2–^ species formed at higher potentials and related to the OER.^29,46^ Here, Ru dissolution appears to be transient, involving not only oxide formation but also subsequent reduction. Possibly, due to the lower number of Ru active sites and tuned electronic properties of Ru atoms in the HEAs, the OER onset potential is shifted to significantly higher values (Figure S11), which makes the UPL of 1.5 V_RHE_ less damaging for the alloyed Ru atoms. After studying the dissolution of Ru during continuous potential cycling, we moved to the potentiostatic electrochemical protocols to separate the anodic and cathodic processes. When the potential of the hold is 1.2 V_RHE_ (Figure 2b,c), Ru dissolves mostly cathodically. However, after the potential of the hold is increased to 1.5 V_RHE_ (Figure S12a), the anodic dissolution increases significantly, balancing the anodic and cathodic contributions in the total dissolution of Ru.

Platinum is the most stable metal throughout the series of the samples. As presented in Figure 2a, Pt dissolution begins with the second CV cycle, which has a UPL of 1.2 V_RHE_. Within this potential limit, primarily, cathodic dissolution is present. Pronounced anodic dissolution is observed only at higher anodic potentials during the third CV cycle, followed by a more intense peak of cathodic dissolution. This agrees with previous studies of Pt dissolution.^25,28,48,49^ However, during potentiostatic holds in Protocols B and C, where the UPLs are 1.2 and 1.5 V_RHE_ (Figures 2b and S12, respectively), Pt dissolution is barely detectable. It is also noteworthy that the onset potential for dissolution is higher for the Pt alloyed in the HEAs compared with pure Pt (Figure S13).

The behavior of rhodium during Protocol A is very similar to that of platinum (Figure 2a). Rhodium starts dissolving anodically when the potential reaches values around 1.1–1.2 V_RHE_ (Figure 2a). These potentials are much higher compared with the onset potential of the dissolution of pure Rh.^29,50,51^ The cathodic dissolution exceeds the anodic dissolution and begins at lower potentials than the cathodic dissolution of Pt but higher potentials than those of Ir and Ru. When anodic and cathodic dissolutions are separated during Protocols B and C, the dominance of cathodic dissolution becomes evident (Figures 2b,c and S12a, respectively).

Overall, alloying of the noble metals in the HEA does not appear to alter the intrinsic dissolution trends of the noble metals. The characteristic cathodic and anodic dissolution peaks are observed in most cases. However, our results indicate that alloying does influence the onset potential of dissolution, shifting the initiation of anodic dissolution processes to higher potentials (Figure S13).

### Calculating Dissolution Potentials

A theoretical understanding of trends in HEA dissolution is important to predict alloy stability and corrosion behavior. Similarities between predicted and experimentally observed trends are of even higher significance. In this work, periodic DFT calculations are used to estimate trends in the thermodynamics of surface alloy dissolution for a model of a single-phase HEA.^52^ We chose fcc-structured HEA phases of elementary composition comparable to that of the experimentally obtained single-phase fcc-HEA, as described in the Experimental Part. The dissolution onset potentials for the metal atoms of each element present in the HEA were calculated by considering two types of atoms with different coordination numbers, which implicitly considered the effect of neighboring atoms when an atom is removed. To simulate the dissolution events, an atom was removed from each generated random configuration, and the resulting structures were fully relaxed (Figure S1). This procedure captures the key local effects, including the influence of the local atomic environment, surface and bulk reconstruction after vacancy formation, and variations in the dissolution stability. However, it does not account for full thermodynamic equilibrium or long-range segregation phenomena, which would require Monte Carlo or molecular dynamics approaches. In comparison to the experimentally determined values shown in Figure 3a, we find that the trend in the onset potential of the individual elements is very well described by the theoretical mean values obtained from the potential distributions of removing a kink or an edge atom, as shown in the inset plots in Figure 3b,c. A comparison of the calculated onset potentials in an HEA compared to the values of the pure metals (given as a line in the corresponding color) is shown in Figure 3b,c. Within our theoretical calculations, platinum, ruthenium, and rhodium are predicted to be more stable in the HEA than in their pure metal forms, whereas the other elements tend to dissolve preferentially at the considered atomic sites. In our experimental study, however, we find that most elements appear to be more stable inside the HEA samples compared to the onset potentials of the pure metals. The DFT calculations rely exclusively on thermodynamically derived equilibrium potentials, which represent an oversimplification of the system but provide insight into the stability and general dissolution trends of the metals and when they are in the HEAs. The discrepancy we find between the dissolution behavior, as predicted from DFT for the idealized metallic surface models versus the experimentally determined, shows that the DFT models do not fully capture the complexity of the catalyst nanoparticle surfaces under experimental conditions, including dynamic surface oxidation and restructuring and effects of the electrochemical environment. On the basis of the differences, we note that these not-considered kinetic effects significantly affect the dissolution behavior of these complex multimetallic particles. A similar observation was made in our previous work.^24^ We want to point out here that there is a need to develop a theory of alloy dissolution that can take other effects into account, aside from thermodynamics.

### Effect of Phase Segregation on Dissolution

Having established the elemental dissolution behavior in a single-phase fcc-HEA, we now examine how phase segregation influences the HEA stability. Overall, the characteristics of the anodic and cathodic dissolution behavior of the individual elements are consistent across the different samples. The dissolution profiles of the 2fcc-HEA and hcp-2fcc-HEA samples recorded during the CV measurements (Protocol A) are presented in Figure S14. The onset potentials of elemental dissolution extracted from these data vary only slightly for the different alloys studied (Figure S13). This indicates that the observed dissolution features are general characteristics of the stability for the studied alloys. Similar trends are observed in the dissolution profiles recorded under Protocols B and C (Figures S15 and S16), further confirming the consistency in anodic and cathodic behavior across the samples. However, the total amount of metal dissolved differs among alloys. To account for compositional differences, we normalize the dissolved metal quantities to the atomic fractions of each element in the alloys, as determined by Protocol A (Figure 4) and compare these across Protocols B and C as well (Figure S17).

**Figure 4:**
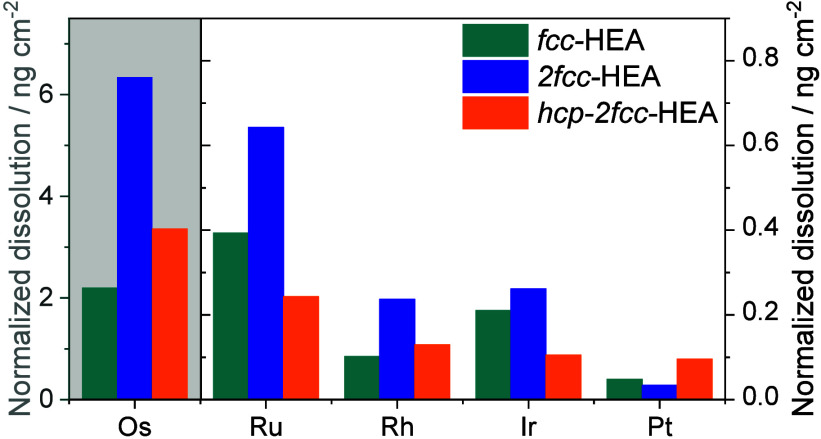
Dissolution of individual elements (normalized to the initial alloy composition) determined from Protocol A..

Because HEAs are very complex multimetallic systems, the alloy with the highest similarity to the fcc-HEA is studied first in detail. The HEA sample that consists of two fcc phases (2fcc-HEA) shares the same elemental composition as the fcc-HEA. As shown in Figure 4, dissolution from the 2fcc-HEA is more pronounced for all elements except platinum. This can be explained by differences in the phase composition and the effects of elemental segregation. While all five elements appear to be evenly distributed in the fcc-HEA, the 2fcc-HEA sample exhibits a high degree of platinum, ruthenium, and osmium separation. This is evident both from the diffraction analysis of the crystallographic phases present and from high-resolution STEM–EDX maps combined with NMF analysis (Figures 1 and S8). The elemental maps reveal regions with significant Pt enrichment, while other areas contain all five elements but with lower platinum concentrations. Because only a small amount of platinum is integrated into the alloy, less platinum is exposed to the electrolyte, leading to reduced Pt dissolution. Additionally, the segregation of Pt reduces its stabilizing interaction with less stable neighboring atoms. As discussed in the previous sections, platinum is the most stable element of the five metals and thus dissolves the least when located in Pt-rich regions. However, when uniformly integrated into an alloy and surrounded by less stable elements, platinum tends to dissolve more due to destabilization effects. The increased dissolution of the other four elements in the 2fcc-HEA, compared to the more homogeneous fcc-HEA, indicates that Pt integration into the alloy has a stabilizing effect on less-stable elements. Another element only partially mixed within the HEA phase is Os (as is evident from the NMF analysis in Figure S9), which exhibits remarkably high dissolution in this sample. This indicates that incomplete mixing of the least stable element, Os, within the structure of the multicomponent alloy promotes its dissolution and might even trigger more rapid dissolution of the other elements. However, direct correlation appears too complex, and we can only make an assumption regarding the mechanism. Possibly, during the exposure to anodic potentials, drastic dissolution of osmium results in exposure of a higher number of Ir, Rh, and Ru atoms to the electrolyte, which leads to their rapid oxidation. Because these elements dissolve cathodically, further reduction of these previously formed oxides triggers the dissolution we observe. All this makes Os the least favorable element for the stability of such alloys. One more feature was revealed by the NMF analysis: the 2fcc-HEA also has local segregation of Ru, which explains its higher dissolution in this alloy (Figures 4 and S17). As mentioned above, ruthenium dissolves mostly cathodically, but at potentials of 1.5 V_RHE_, anodic dissolution starts to play a significant role (Figures S15 and S16). In Protocol C, Ru dissolution is higher than that in Protocol B (Figure S17). One can also notice that when Ru is alloyed with more stable elements like Pt, its exposure to high anodic potentials does not lead to such a dramatic increase in dissolution: Ru dissolution in the fcc-HEA in Protocol C is less than 2 times higher than that in Protocol B, while its dissolution in 2fcc-HEA and hcp-2fcc-HEA is approximately 3 times higher.

We also compare the amounts of metals dissolved from the hcp-2fcc-HEA sample. In this case, the amounts of dissolved iridium, as well as ruthenium, are lower than those in the single-phase fcc-HEA. Pt, Rh, and Os, however, dissolve in larger amounts in the hcp-2fcc-HEA sample. Considering the dissolution behavior of the individual elements, this corresponds to an increased anodic and a less pronounced cathodic dissolution than those in the single-phase fcc-HEA. The hcp-2fcc-HEA sample is characterized by a high degree of element segregation: three separate crystallographic phases occur in the diffraction pattern. On the local level, elemental segregation is also apparent but less pronounced. Through NMF analysis of the STEM–EDX maps, we found that areas containing Os, Ru, and Rh are free of Pt and Ir, while Pt-rich areas contain lower amounts of Rh and Os. The fact that cathodically corroding elements like Ru and Ir exhibit improved stability in this sample than in the fcc-HEA highlights that dissolution behavior is strongly dependent on the phase composition. This underscores that achieving crystallographic phase purity in HEA nanomaterials does not necessarily result in a maximum elemental stability. Instead, local atomic coordination and the spatial distribution of elements within individual nanoparticles play critical roles.

The dissolution behavior of these five elemental HEA samples appears to be complex, and many factors influence the comparison of different multimetallic alloys. Even samples that differ by only one synthesis parameter can exhibit different degrees of element dissolution. This is most likely related to local atomic coordination, specifically, which neighboring atoms surround each element. Well-mixed distributions, such as those between Ir and Ru (both highly active OER catalysts), may help to suppress their cathodic corrosion. Meanwhile, the inclusion of Pt tends to stabilize surrounding elements, but, on the other hand, those elements can destabilize Pt, resulting in higher dissolution.

At this point in our work, it is extremely challenging to draw conclusions about the contribution of every phase to the overall dissolution of the alloys, build a model, or predict behavior under different electrochemical conditions. More work needs to be done to improve our understanding of noble-metal dissolution from various crystal structures in such multielemental alloys. This work can serve as a starting point for further examination of the stability of the noble-metal HEAs. Moreover, although Os plays a destabilizing role, one can substitute it with metals like Au or even non-noble metals (e.g., Ni, Co, or Fe) to further explore the stabilizing (or destabilizing) effect of certain metals and how their dissolution behavior changes depending on the crystal phase, local environment, and neighboring metals.

## Conclusions

Our study shows that the dissolution behavior of five elemental HEA samples with similar overall compositions is strongly influenced by elemental mixing and local atomic coordination. This underscores the importance of detailed structural characterization when attempting to establish structure–property relationships in these complex materials. Our results indicate that elemental mixing and single-phase HEA nanoparticles promote stability and lessen dissolution. Also, the inclusion of stable elements, such as Pt, significantly influences the overall dissolution behavior. More broadly, our results demonstrate that the stability of these complex alloys is highly dependent on the applied electrochemical protocols. Therefore, beyond thermodynamic considerations, the HEA design should include the reaction conditions usually applied in a given application. To design multimetallic alloys with tailored electrocatalytic properties, new theoretical models that go beyond thermodynamics must be developed to guide targeted materials design.

## Supplementary Material



## Notes

Calculations and structural characterization of the prepared materials, Rietveld refinement results, NMF analysis of the STEM–EDX data of the HEAs, elemental mappings, TEM images of the HEA samples, cyclic voltammograms, and additional dissolution profiles and analysis (PDF)

## ‡

Department of Chemistry and Chemical Biology, Baker Lab, Cornell University, Ithaca, NY 14853, USA

## §

Catalysis Theory Center, Department of Physics, Technical University of Denmark, Kongens, Lyngby 2800, Denmark.
