# Supplementary material for: Effect of Alloying and Phase Segregation on the Stability of High-Entropy Alloys: A Case Study on the Dissolution of Os–Ru–Rh–Ir–Pt Nanoparticles
Source: ACS Appl Eng Mater. 2026 Jun 11;4(7):3727–37. doi: 10.1021/acsaenm.6c00379 (PMC13411044; doi:10.1021/acsaenm.6c00379)
Supplement: Supplementary file 1 [file em6c00379_si_001.pdf]

Supporting Information

The Effect of Alloying and Phase Segregation on the Stability of High Entropy Alloys: A Case Study on the Dissolution of Os-Ru-Rh-Ir-Pt Nanoparticles

Tatiana Priamushko,[1,,a] Rebecca K. Pittkowski,[2,*,a] Maria Minichova,[1,3] Attila Kormányos,[4] Valentin Briega-Martos,[1,†] Luis A. Cipriano,[2,†] Nicolas Schlegel,[2,5] Rasmus Rohde,[6] Espen D. Bøjesen,[6] Kirsten M. Ø. Jensen,[2] Jan Rossmeisl,[2] Matthias Arenz[5,*] Serhiy Cherevko[1,*]*

[1] Forschungszentrum Jülich GmbH, Helmholtz Institute Erlangen-Nürnberg for Renewable Energy (IET-2), Cauerstr. 1, 91058 Erlangen, Germany

[2] Center for High Entropy Alloy Catalysis (CHEAC), Department of Chemistry, University of Copenhagen, 2100 Copenhagen Ø, Denmark

[3] Institute of Chemical Reaction Engineering, Friedrich-Alexander-Universität Erlangen-Nürnberg, Egerlandstr. 3, 91058 Erlangen, Germany

[4] Department of Physical Chemistry and Materials Science, University of Szeged, H-6720, Szeged, Hungary

[5] Department of Chemistry, Biochemistry and Pharmaceutical Sciences, University of Bern, 3012 Bern, Switzerland

[6] Center for Sustainable Energy Materials (CENSEMAT) & Interdisciplinary Nanoscience Center, Aarhus University, Aarhus, Denmark

[a] T.P. and R.K.P. contributed equally to this work.

[†] Present addresses: V. B.-M.: Department of Chemistry and Chemical Biology, Baker Lab, Cornell University, Ithaca, NY 14853, USA, L. A. C.: Catalysis Theory Center, Department of Physics, Technical University of Denmark, Kongens Lyngby 2800, Denmark

*Corresponding authors: tatyana.pryamushko@gmail.com, rebecca.pittkowski@chem.ku.dk, matthias.arenz@unibe.ch, s.cherevko@fz-juelich.de

Part A – Calculations & Structural Characterization of the prepared materials

Theoretical simulations of OsRuRhIrPt HEAs surfaces

As mentioned in the main text, hundreds of random OsRuRhIrPt HEA surfaces were generated by the average lattice parameter of the DFT calculated bulk metals. However, in the main text, it does not explicitly describe how these structures were generated. First, for each kink or edge surface, 100 random structures were generated. Subsequently, the kink or edge atoms in these surfaces were replaced by an Os, Pt, Ru, Rh, or Ir atom, with a total of 1000 structures for both surfaces. Afterward, the replaced atoms were removed, and the cohesive energy of removing the atoms from the HEAs was calculated (see below). A scheme representation of the atom dissolution is shown in Figure S1, and Figure S2 shows the side and top views of the kink and edge surfaces.

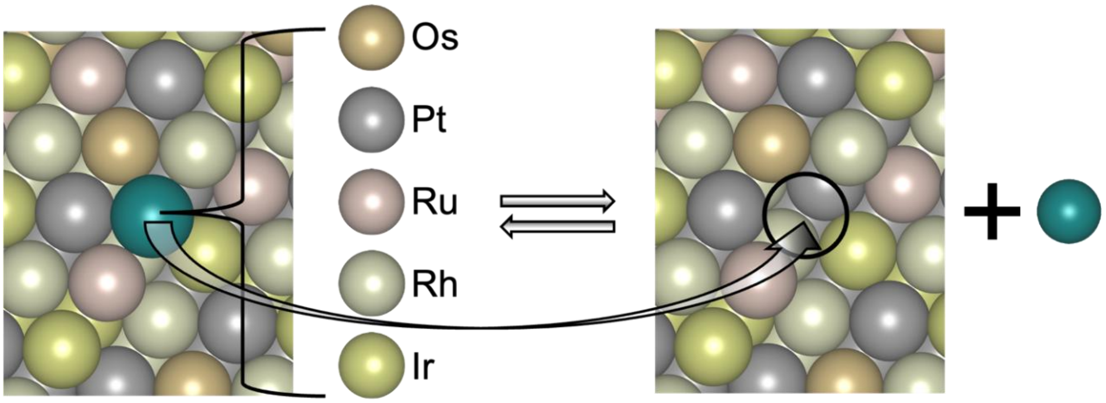

Figure S1. *Dissolution scheme on an edge atom from the OsRuIrPtRh HEAs. The same scheme was used for the dissolution of kink atoms.*

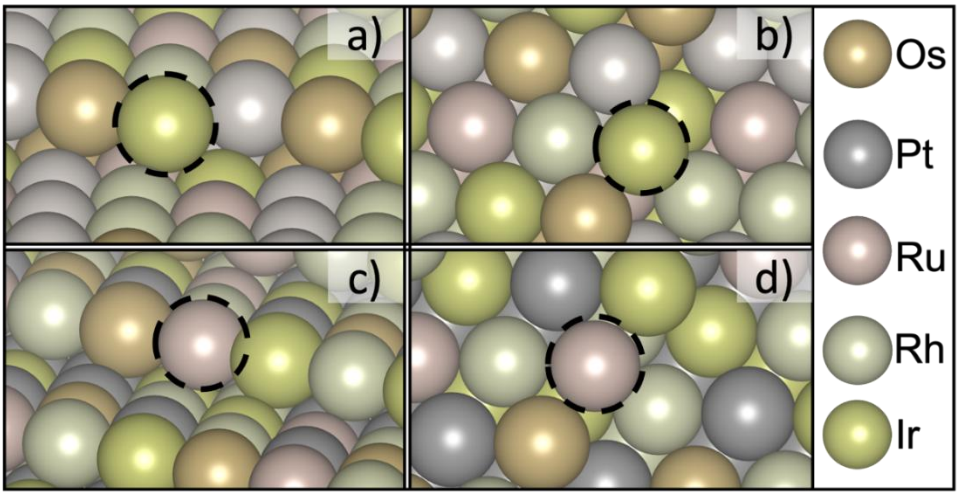

***Figure S2**. a) side and b) top view of the Kink OsRuRhIrPh HEA surface used to simulate the dissolution of kink atoms. c) side and d) top views of the Edge OsRuRhIrPt HEA surface used to simulate the dissolution of edge atoms. The Kink and Edge atoms are indicated by a dashed black line.*

Standard electrode potentials

The theoretical onset potentials (U_{diss}) for each type of atom were calculated by computing the cohesive energy of removing an atom from the randomly generated OsRuIrPtRh HEA surfaces with equation (1), see also Figure S1.

$$\Delta E_{DFT} = (E_{HEA-M} + E_{M,Bulk}) - E_{HEA} \qquad \text{(eqn S1)}$$

With ΔE_{DFT} as the cohesive energy of dissolving a kink or edge atom from the HEA. E_{HEA} is the energy of the HEA surface for each type of atom, $E_{M,Bulk}$ is the bulk energy of the removed metal, and E_{HEA-M} is the energy of HEA surface without a kink or edge atom (M = Os, Ru, Ir, Pt or Rh).
Subsequently, these energies were converted into potentials by using equation 2 and by assuming that the dissolved ions will be in the oxidation state that leads to the most thermodynamically favorable dissolution events according to the electrochemical series (versus the Standard Hydrogen Electrode– SHE).

$$U_{diss} = \frac{\Delta E_{DFT}}{ne} + U_M^{\circ} \qquad \text{(eqn S2)}$$

Where U_{diss} is the expected onset potential for the dissolution of a kink or edge atom from the HEA. ΔE_{DFT} is the cohesive energy of removing a kink or edge atom from the HEA, ne is the number of electrons transferred during the dissolution process (M = Os, Ru, Ir, Pt or Rh), and U_M° is the standard electrode potential of pure metal versus SHE (Table S1).

Table S1. Considered reactions to calculate the theoretical onsets and their corresponding standard electrode potentials.

$Pt^0 \leftrightarrow Pt^{2+} + 2e^-$	$E° = 1.18$ V
$Ir^0 \leftrightarrow Ir^{3+} + 3e^-$	$E° = 1.16$ V
$Os^0 + 2\ H_2O \leftrightarrow OsO_2 + 4H^+ + 4e^-$	$E° = 0.69$ V
$Rh^0 \leftrightarrow Rh^{3+} + 3e^-$	$E° = 0.80$ V
$Ru^0 + 4\ H_2O \leftrightarrow RuO_4^{2-} + 8H^+ + 6e^-$	$E° = 1.19$ V

Rietveld refinements

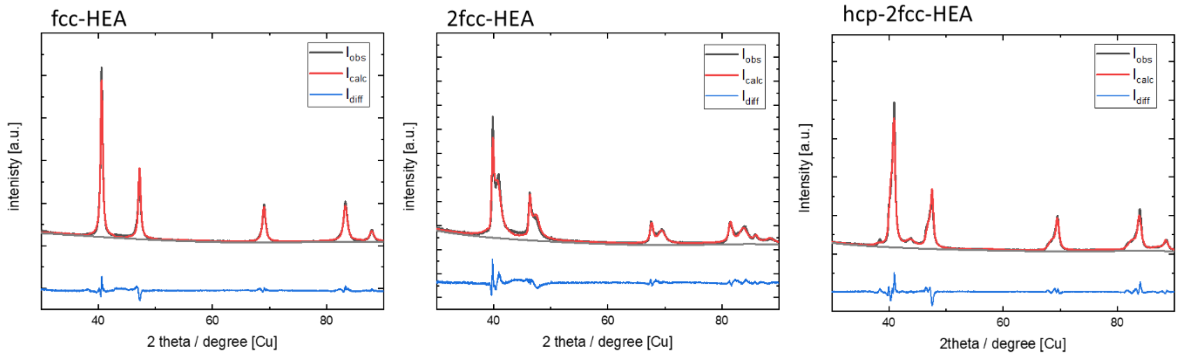

Figure S3. Rietveld refinement of the HEAs

Table S2. Results of Rietveld refinement of the diffraction patterns to quantify the crystallographic phases present in the different HEA samples.

**Sample**	***fcc-HEA***	***2fcc-HEA***	***hcp-2fcc-HEA***
Phases	1 fcc	2 fcc	1hcp + 2fcc
R_{wp}	11.5	16.0	12.8
Phase (fraction)		*fcc1 (60 wt.%)*	*fcc1 (61 wt.%)*
Lattice parameters, Å	*a*=3.8458(1)	*a*=3.8332(3)	*a*=3.8294(2)
Phase (fraction)		*fcc2 (40 wt.%)*	*fcc2 (25 wt.%)*
Lattice parameters, Å		*a*=3.9202(2)	*a*=3.8792(4)
Phase (fraction)			*hcp (14 wt.%)*
Lattice parameters, Å			*a*=2.7059(1),
			c=4.3770(5)

NMF analysis of STEM-EDX data

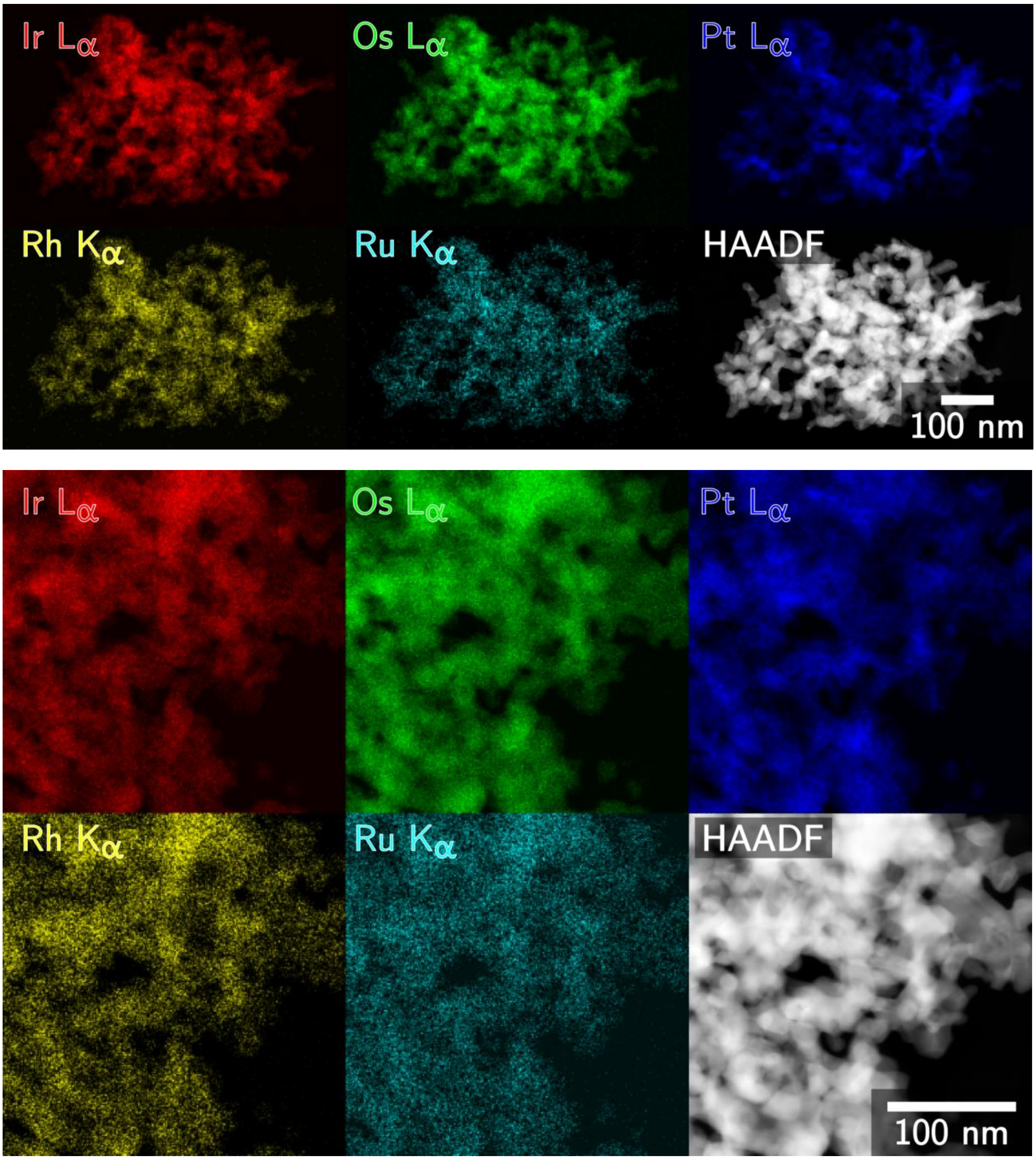

Figure S4. Elemental mappings of the *fcc*-HEA sample.

All elements except Pt appear to be uniformly mixed, as indicated by the elemental mapping images presented in **Figure S4**. Further analysis, using non-negative matrix factorization (NMF), revealed some local inconsistencies in the chemical composition of the alloys. **Figure S5** demonstrates the EDX elemental mapping images of three selected factors in red, green, and blue with the respective EDX spectra below. The red factor contains a Pt-rich mix of all elements, whereas the green factor is Ir-rich but contains no Pt, and the blue factor is rich in Os and Rh but has no Ir or Pt. We can assume that these three components are similar to each other in terms of the crystal structure and are relatively well mixed, as the XRD pattern did not show any obvious phase segregation. Importantly, no segregation of single metals was observed in this sample, which means that all the elements are most likely present in at least tertiary alloys.

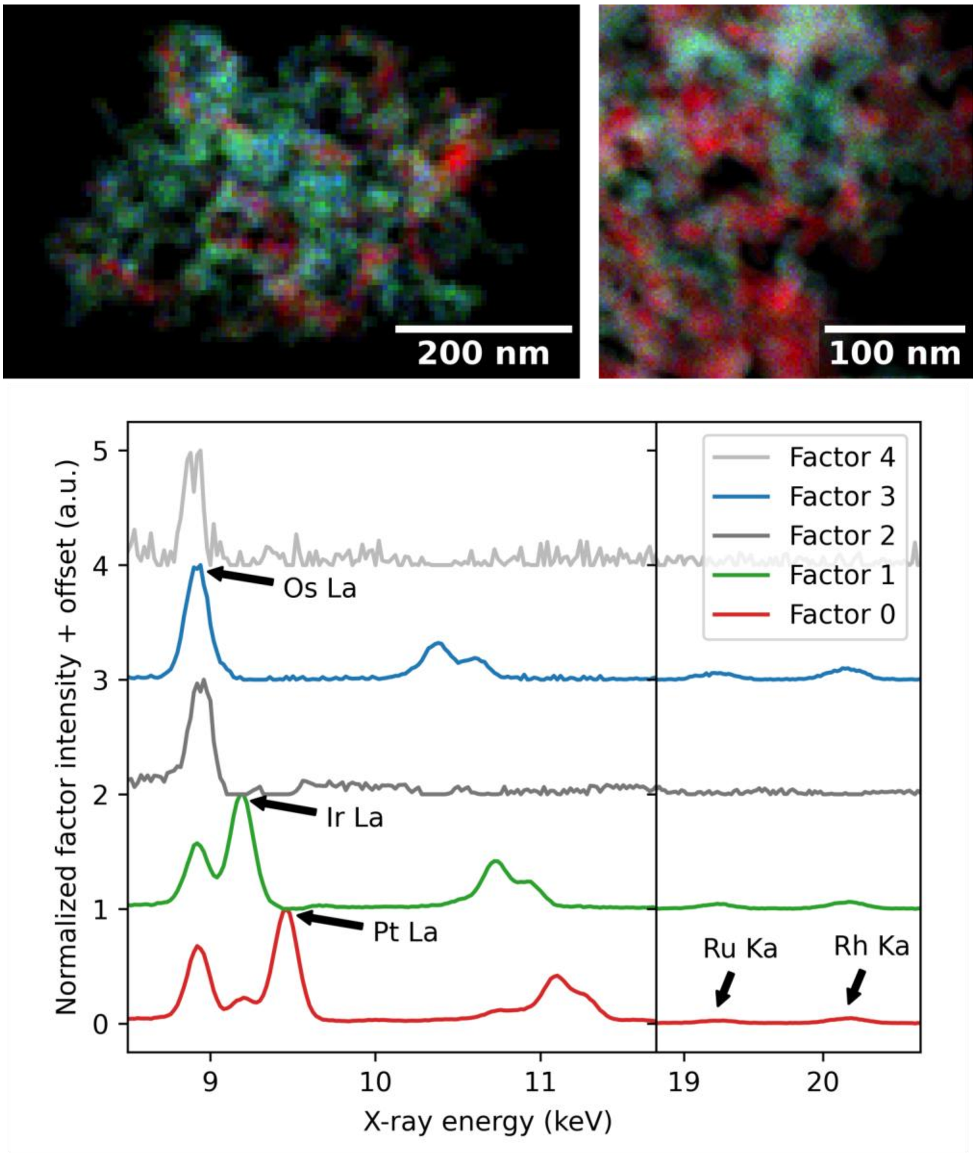

Atomic percentages (~ ± 5 at%)					
**Factor**	**Os**	**Ru**	**Ir**	**Pt**	**Rh**
**0**	25	8	8	40	19
**1**	21	14	39	0	26
**3**	36	21	1	0	42

Figure S5. Elemental mappings (loadings) and their NMF components (factors) of the *fcc*-HEA sample. The red, green, and blue colors in the mappings are factors 0, 1, and 3, respectively. Factors 2 and 4 were left out for being unphysical. The atomic percentages were found by Cliff—Lorimer quantification using the X-ray peak intensities by fitting the peaks indicated with arrows with Gaussians on a linear background. It is important to note that the factors from the NMF are not necessarily real chemical phases, but mathematical representations of the EDX data that can change drastically depending on e.g. the binning of the data or the chosen number of components. Additionally, Cliff—Lorimer quantification has a high uncertainty, but it is often around 5 at%.

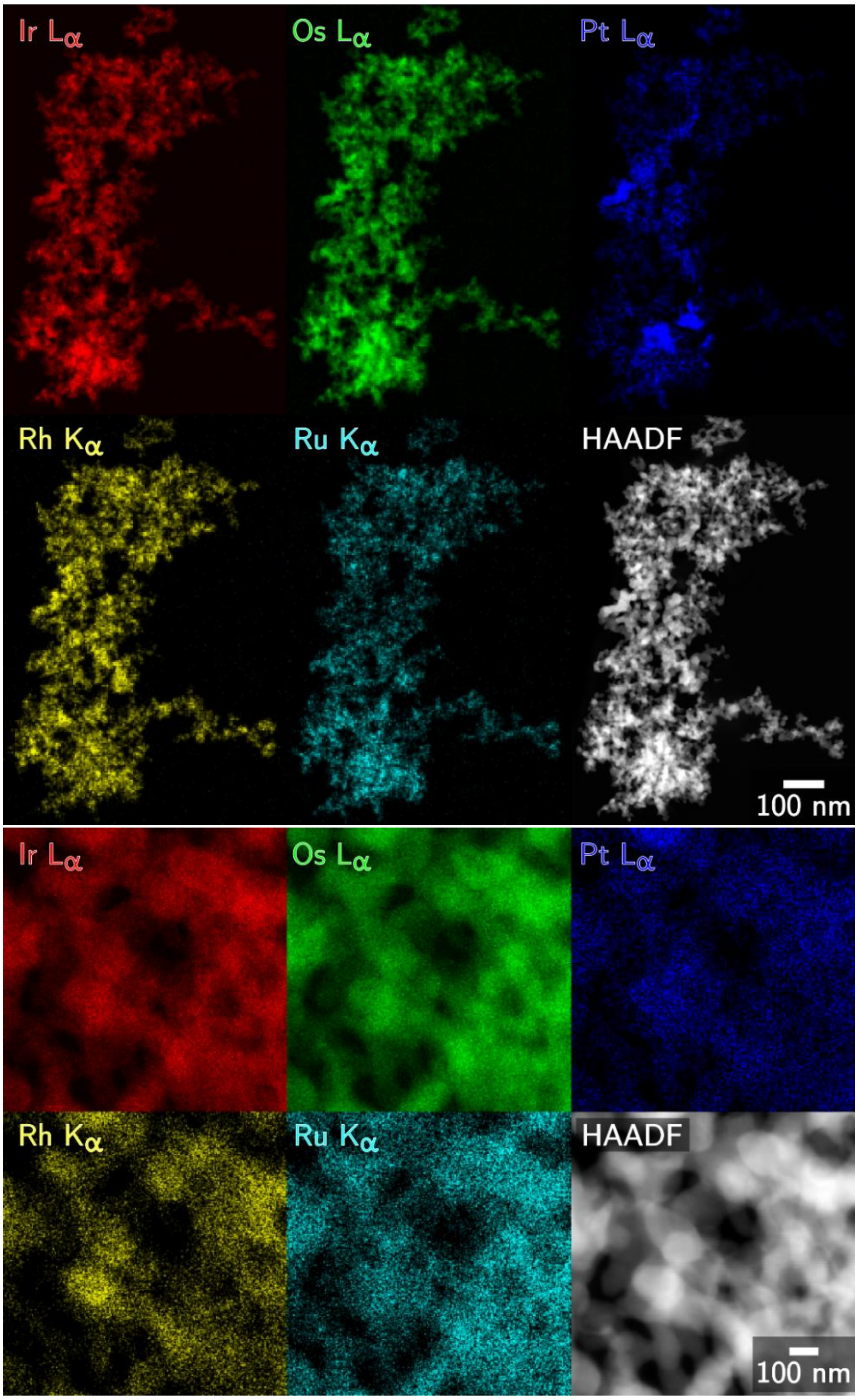

Figure S6. Elemental mappings of the 2*fcc*-HEA sample.

Figure S6 presents additional elemental mapping images of the 2*fcc*-HEA sample. As can be seen in the first set of images, Pt-enriched and Pt-poor areas are present in this sample. From the Rietveld refinement of the structure based on the XRD analysis, we assumed that the second *fcc* phase might be assigned to the partially segregated Pt. On the other hand, all the other elements seem to be distributed evenly. Surprisingly, the NMF components analysis suggests that both Os and Pt are not fully mixed into the structure of the alloy. **Figure S7** shows three NMF components colored in red, green, and blue, corresponding to the EDX spectra below. The red factor contains all elements but Pt, the green mostly Os, and the blue much Rh but no

Ru. However, the images show that the red and green components are rather well mixed (red + green = yellow), indicating that it is mostly the Pt-rich component that segregates.

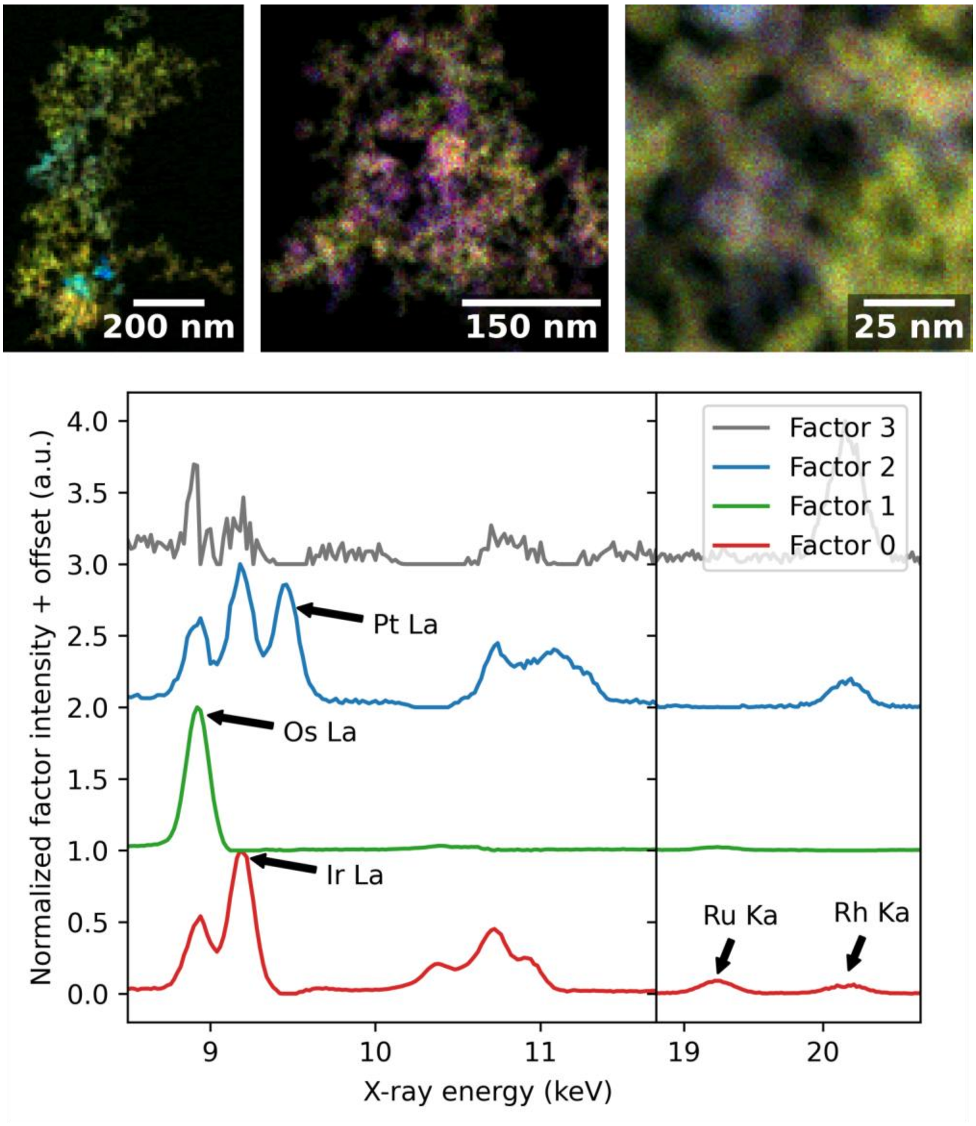

Atomic percentages (~ ± 5 at%)					
**Factor**	**Os**	**Ru**	**Ir**	**Pt**	**Rh**
**0**	17	27	33	0	23
**1**	83	17	0	0	0
**2**	13	0	21	19	47

Figure S7. Elemental mappings and their NMF components of the 2*fcc*-HEA sample. Factors 0, 1, and 2 are colored in red, green, and blue, respectively. Factor 3 is unphysical and was left out. The yellow color in the images is a result of a green and red color combination. The atomic percentages were found as described in **Figure S5**.

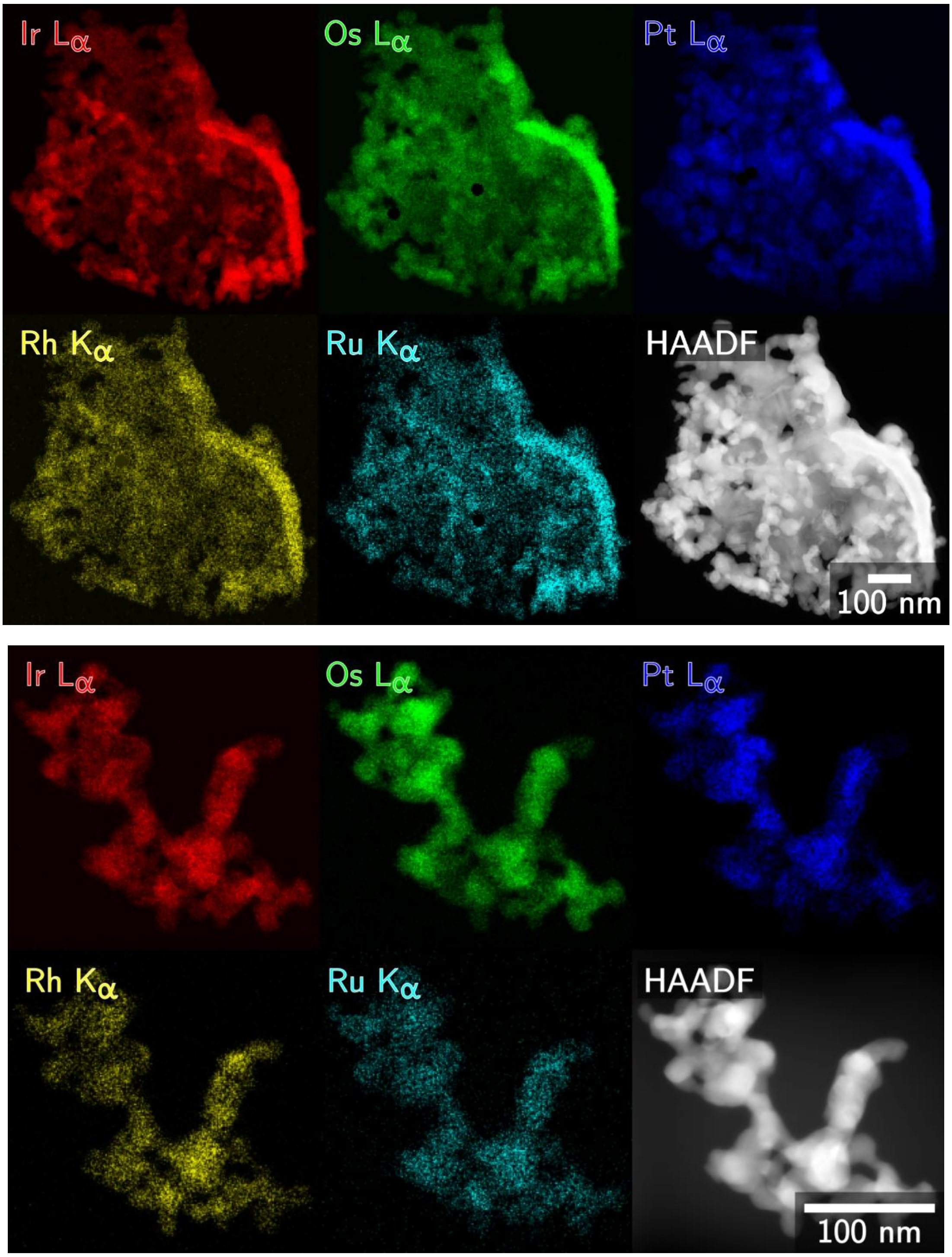

Figure S8. Elemental mappings of the *hcp*-2*fcc*-HEA sample.

According to **Figure S8**, all the elements except Pt in the *hcp*-2*fcc*-HEA sample are distributed evenly and are well mixed. However, both the XRD analysis (**Figure 1a-b**) and the NMF analysis (**Figure S9**) indicate phase segregation in this material. The red color in **Figure S9** represents the EDX spectrum of the Os-, Ru- and Rh-rich factor 0. The green and blue are Pt-rich factor 1 and Ir-rich factor 2 respectively. The images show that while the red and blue components are rather well mixed, the Pt-rich blue component segregates. Although it is not clear what phases are formed by which elements, such analysis of the NMF components is very useful for our dissolution study, as it can help with interpreting the varied stability of the elements and trends in dissolution.

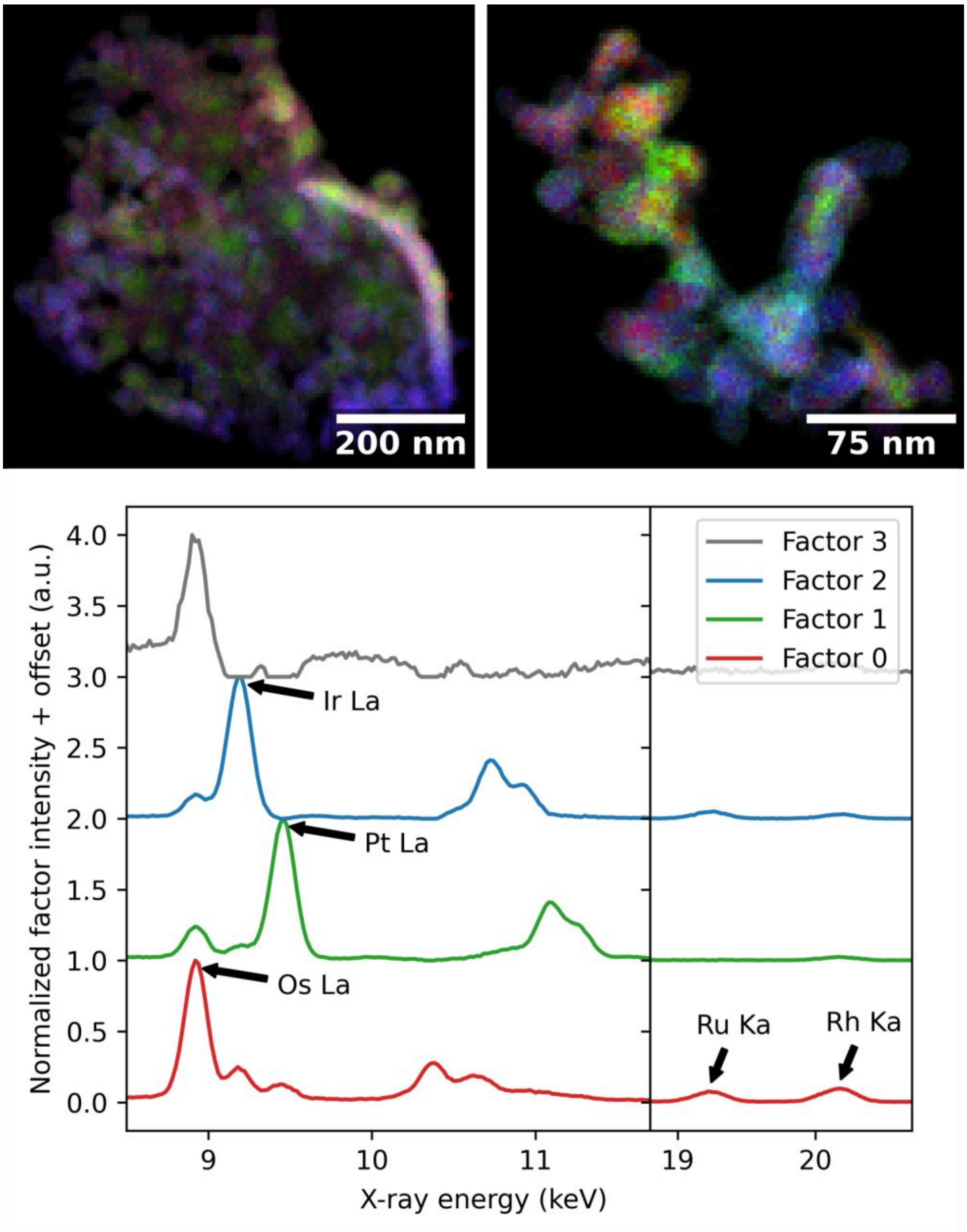

Atomic percentages (~ ± 5 at%)					
**Factor**	**Os**	**Ru**	**Ir**	**Pt**	**Rh**
**0**	32	21	7	4	36
**1**	13	0	6	64	17
**2**	8	23	52	0	17

Figure S9. Elemental mappings and their NMF components of the *hcp*-2*fcc*-HEA sample. Factors 0, 1, and 2 are colored in red, green, and blue, respectively. Factor 3 is unphysical and was left out. The purple color in the images is a result of a red and blue color combination. The atomic percentages were found as described in **Figure S5**.

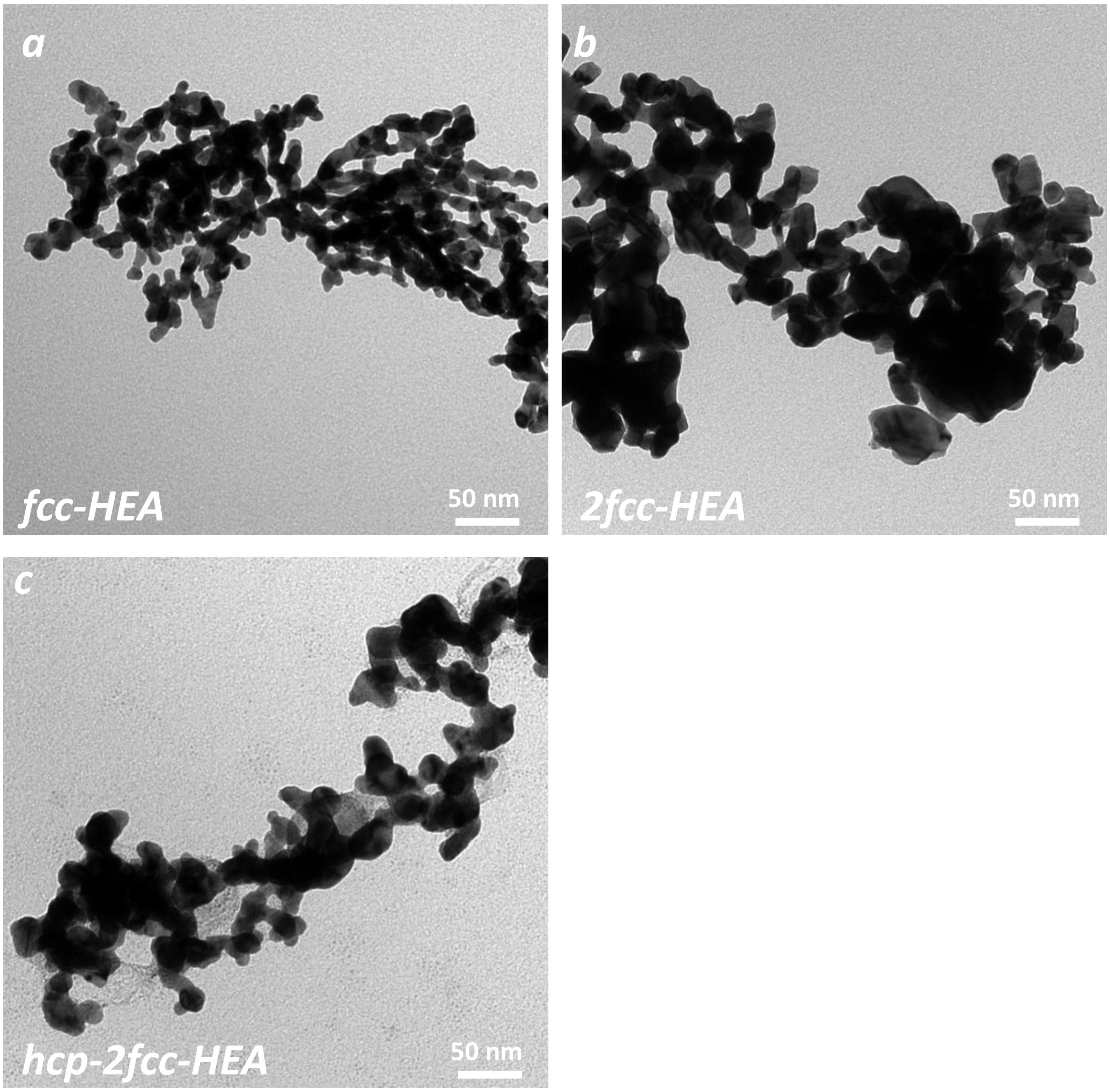

Figure S10. Selected TEM micrographs of the three HEA samples analyzed in this study; *fcc*-HEA, b) *2fcc*-HEA, and c) hcp-2fcc-HEA, highlighting the intergrown particle morphology of the samples.

Part B – Electrochemical characterization & dissolution studies

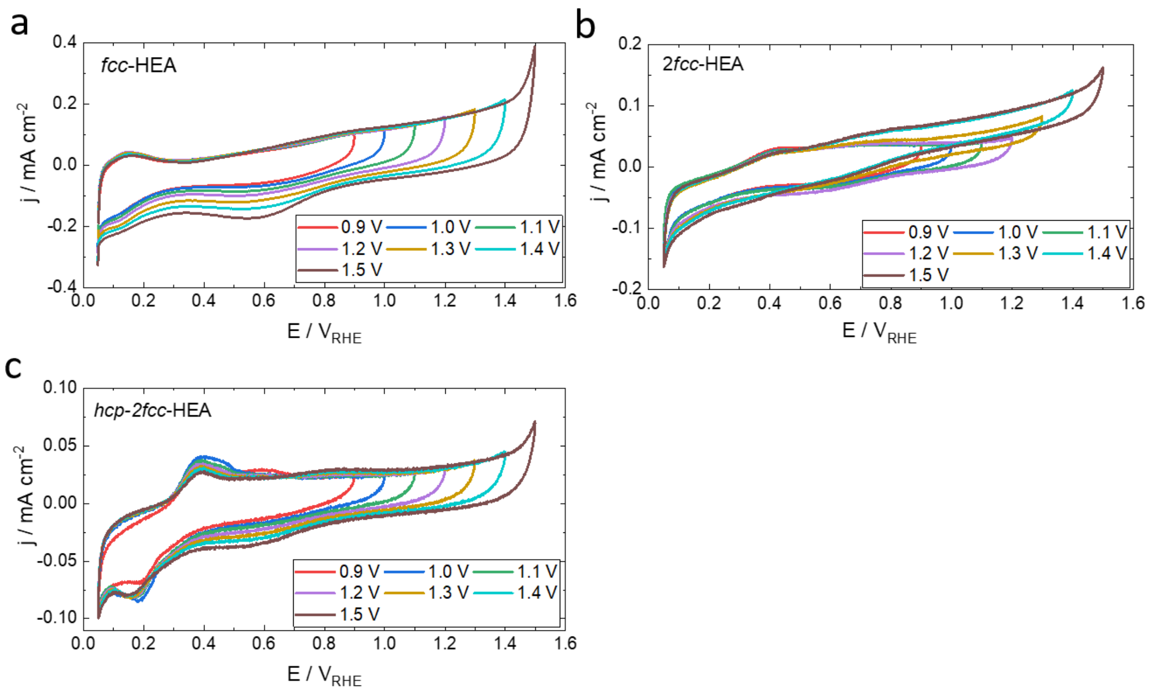

Figure S11. Cyclic voltammograms of HEAs recorded in 0.1 M $HClO_4$ for the a) *fcc*-HEA, b) *2fcc*-HEA, and c) hcp-2fcc-HEA sample

General electrochemical behavior. Traces of oxygen are visible in the shape of the CVs. Although a detailed analysis of the cyclic voltammetry profile is complex due to the large number of elements involved, with different ratios in each HEA, some general behaviors can be identified. The *fcc*-HEA shows the most Pt-like behavior, as it is the most abundant in this case, with a clear hydrogen adsorption/desorption region and an oxide reduction peak at a potential similar to that of pure Pt. For the *2fcc*-HEA, a similar voltammetric profile would be expected, but probably the fact that Pt is segregated makes the features of the other elements more noticeable since their active sites would be less affected by Pt. For the *hcp-2fcc*-HEA, the cathodic peak at ca. 0.15 V can probably be ascribed to Rh sites, while the anodic peaks at ca. 0.5 V might be related to both Rh and Ir.

Dissolution of a single-phase fcc high-entropy alloy

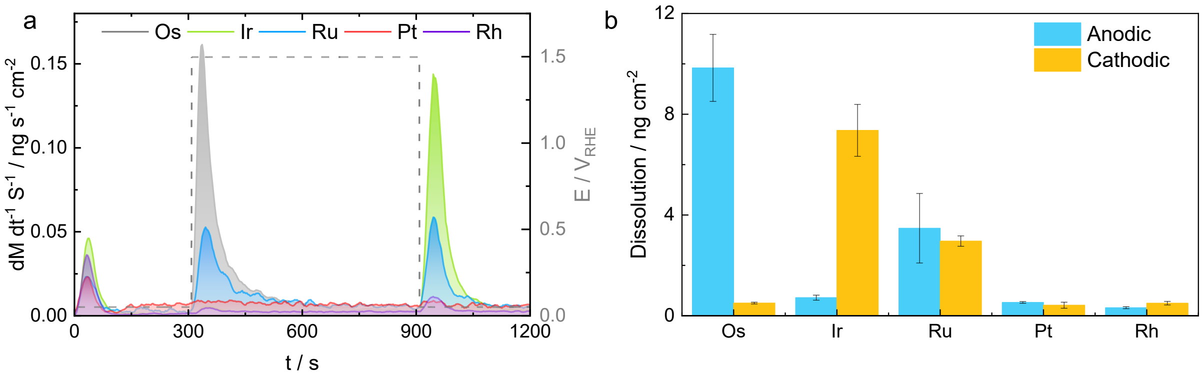

Figure S12. Dissolution profiles by element of the *fcc*-HEA sample as observed by a) *Protocol C* and b) dissolved amounts of metals extracted from the integrated anodic and cathodic dissolution peaks.

Dissolution of phase-segregated high-entropy alloys

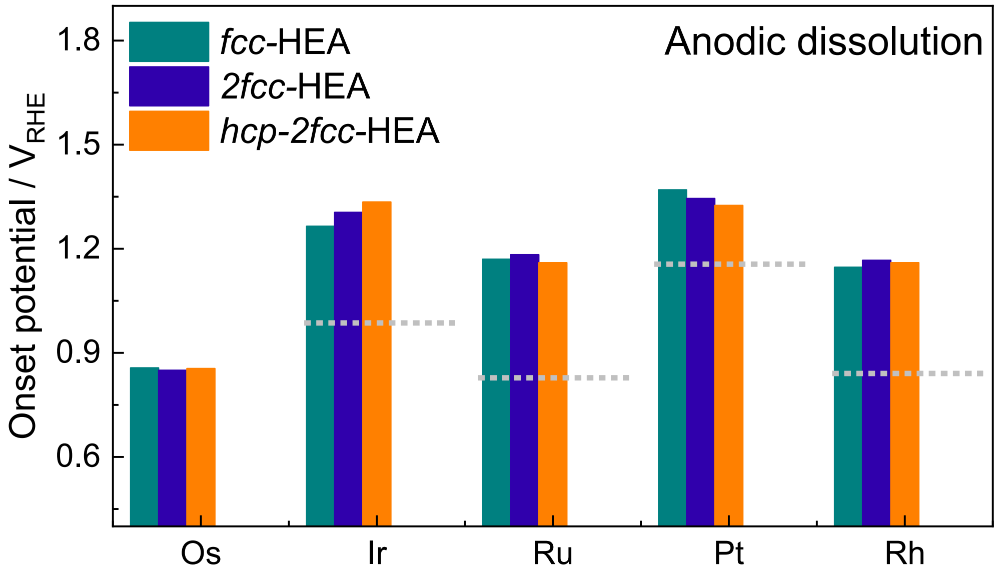

Figure S13. Comparison of the dissolution onset potential of the HEAs. The dashed lines refer to the onset potential of dissolution observed for the thin films' single-element samples in the previous study.[1]

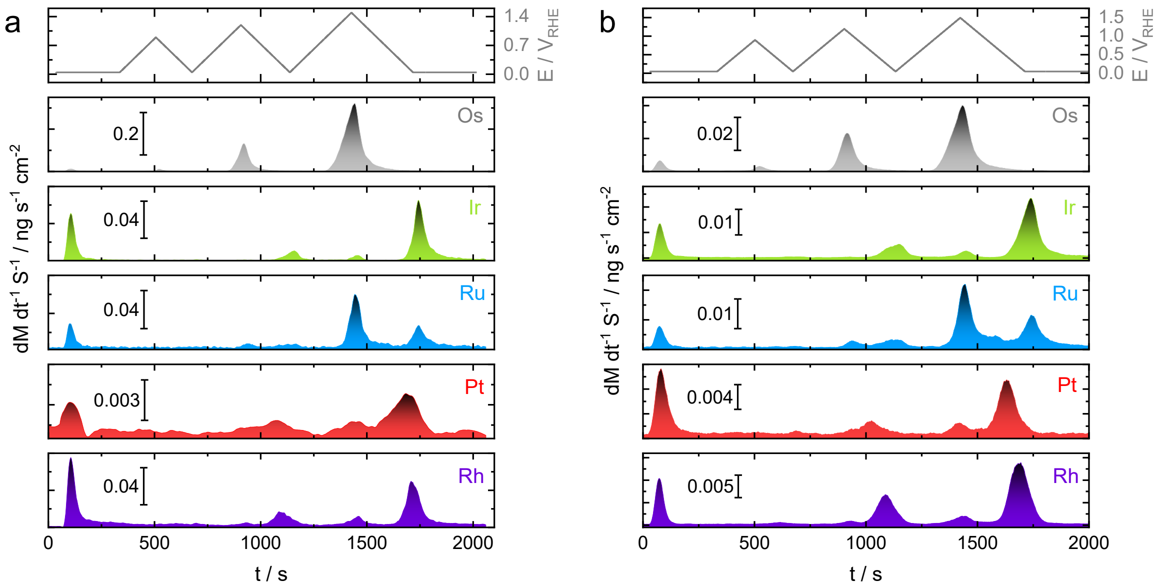

Figure S14. Dissolution profiles by element of the a) *2fcc*-HEA and c) *hcp-2fcc*-HEA samples as observed by *Protocol A*.

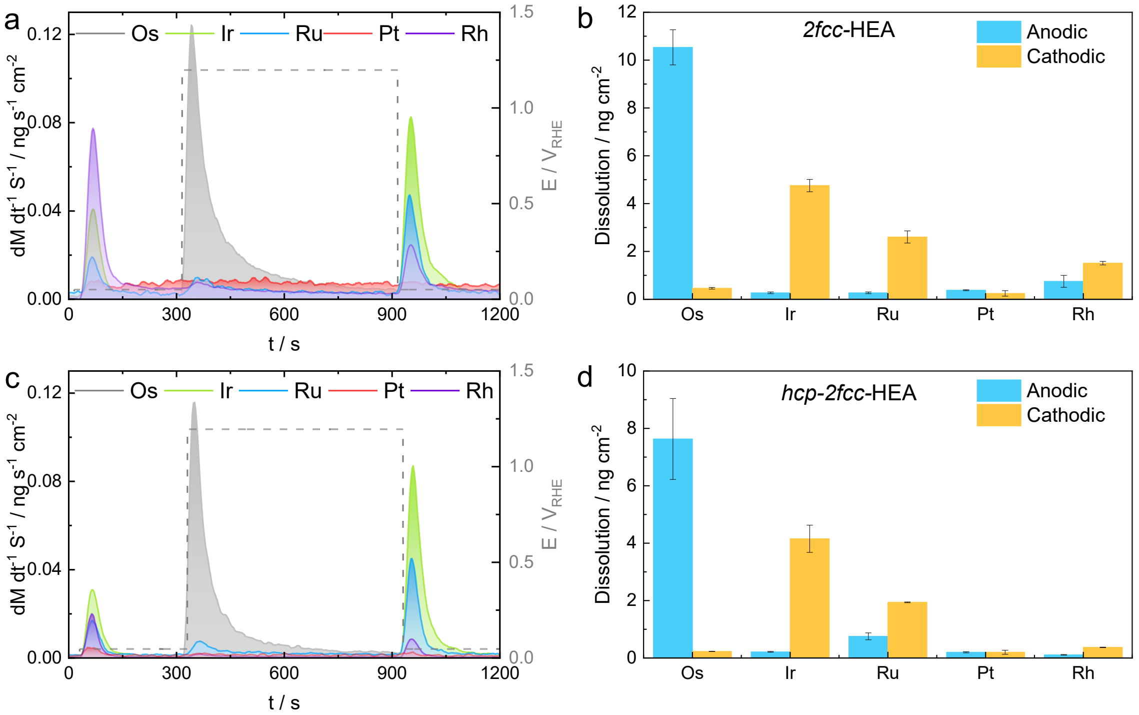

Figure S15. Dissolution profiles by element of the a) *2fcc*-HEA and c) *hcp-2fcc*-HEA samples as observed by *Protocol B.* Dissolved amounts of metals extracted from the integrated anodic and cathodic dissolution peaks of b) *2fcc*-HEA and d) *hcp-2fcc*-HEA.

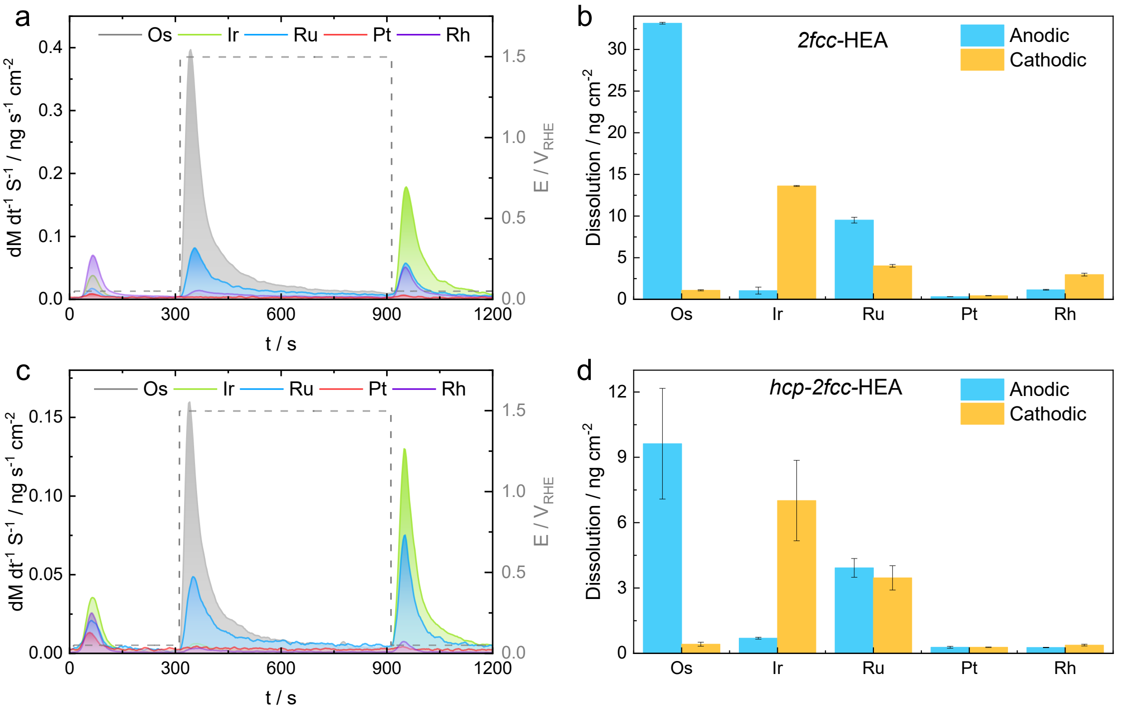

Figure S16. Dissolution profiles by element of the a) *2fcc*-HEA and c) *hcp-2fcc*-HEA samples as observed by *Protocol C.* Dissolved amounts of metals extracted from the integrated anodic and cathodic dissolution peaks of b) *2fcc*-HEA and d) *hcp-2fcc*-HEA.

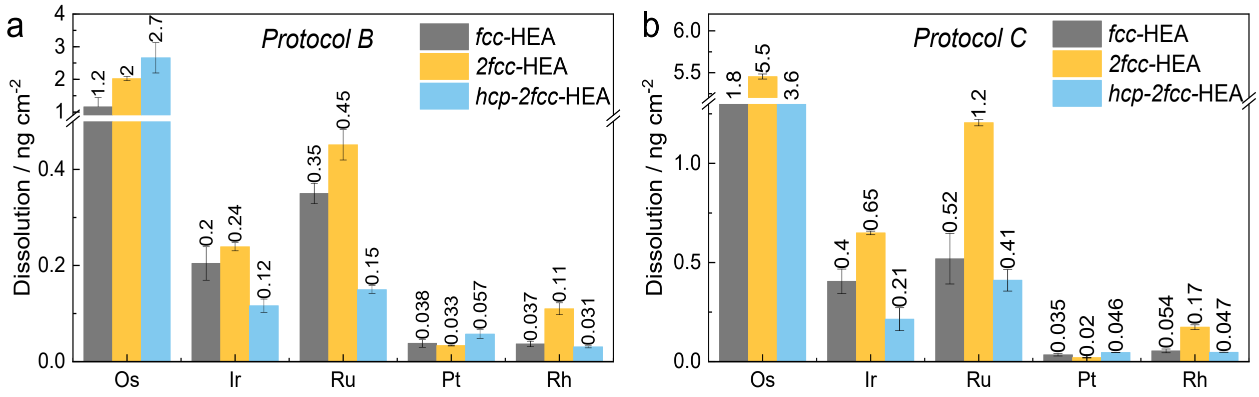

Figure S17. Dissolution of individual elements (normalized to the initial alloy composition) determined from a) *Protocol B* and b) *Protocol C.*

References

[1] Kormányos, A., Dong, Q., Xiao, B., Li, T. Y., Savan, A., Jenewein, K., Priamushko, T., Körner, A., Böhm, T., Hutzler, A., Hu, L. B., Ludwig, A., Cherevko, S. *Iscience* **2023**, *26*.
